# Regulation of locomotion and motoneuron trajectory selection and targeting by the *Drosophila* homolog of Olig family transcription factors

**DOI:** 10.1016/j.ydbio.2012.06.027

**Published:** 2012-09-15

**Authors:** Justine Oyallon, Holger Apitz, Irene Miguel-Aliaga, Katarina Timofeev, Lauren Ferreira, Iris Salecker

**Affiliations:** a Division of Molecular Neurobiology, MRC National Institute for Medical Research, London, NW7 1AA, UK; b Department of Zoology, University of Cambridge, Cambridge, CB2 3EJ, UK

**Keywords:** bHLH transcription factor, Neuron specification, Glial development, Motoneurons, Locomotion

## Abstract

During the development of locomotion circuits it is essential that motoneurons with distinct subtype identities select the correct trajectories and target muscles. In vertebrates, the generation of motoneurons and myelinating glia depends on Olig2, one of the five Olig family bHLH transcription factors. We investigated the so far unknown function of the single *Drosophila* homolog Oli. Combining behavioral and genetic approaches, we demonstrate that *oli* is not required for gliogenesis, but plays pivotal roles in regulating larval and adult locomotion, and axon pathfinding and targeting of embryonic motoneurons. In the embryonic nervous system, Oli is primarily expressed in postmitotic progeny, and in particular, in distinct ventral motoneuron subtypes. *oli* mediates axonal trajectory selection of these motoneurons within the ventral nerve cord and targeting to specific muscles. Genetic interaction assays suggest that *oli* acts as part of a conserved transcription factor ensemble including Lim3, Islet and Hb9. Moreover, *oli* is expressed in postembryonic leg-innervating motoneuron lineages and required in glutamatergic neurons for walking. Finally, over-expression of vertebrate Olig2 partially rescues the walking defects of *oli*-deficient flies. Thus, our findings reveal a remarkably conserved role of *Drosophila* Oli and vertebrate family members in regulating motoneuron development, while the steps that require their function differ in detail.

## Highlights

► *Drosophila* Oli is expressed in embryonic ventral motoneuron subtypes. ► *oli* controls axonal trajectory selection and muscle targeting during embryogenesis. ► Oli acts as part of a conserved transcription factor ensemble that includes Hb9. ► Oli is expressed in postembryonic leg-innervating motoneuron lineages. ► Oli is required in glutamatergic neurons for adult locomotion.

## Introduction

The generation of coordinated muscle contractions, enabling animals to perform complex movements, depends on the assembly of functional neuronal motor circuits. Motoneurons lie at the heart of these circuits, receiving sensory input directly or indirectly via interneurons within the central nervous system (CNS) and relaying information to muscles in the periphery. During development neural precursors give rise to progeny that eventually adopt unique motoneuron subtype identities (Dalla Torre di Sanguinetto et al., 2008; Dasen, 2009). Their axons each follow distinct trajectories into the periphery to innervate specific target muscles. Our understanding of the molecular mechanisms that control the differentiation and respective connectivity of distinct neuronal subtypes is still limited.

The Olig family of basic Helix–Loop–Helix (bHLH) transcription factors in vertebrates includes the Oligodendrocyte lineage proteins Olig1–3, Bhlhb4 and Bhlhb5 (Bertrand et al., 2002). All members play pivotal roles in regulating neural development. Olig2 controls the sequential generation of somatic motoneurons and one type of myelinating glia, the oligodendrocytes, from the pMN progenitor domain in the ventral neural tube (Lu et al., 2002; Lu et al., 2000; Mizuguchi et al., 2001; Novitch et al., 2001; Zhou and Anderson, 2002; Zhou et al., 2001; Zhou et al., 2000). Olig2 mediates progenitor domain formation by cross-repressive transcriptional interactions (Briscoe and Novitch, 2008; Dessaud et al., 2007) and motoneuron differentiation upstream of the LIM-homeodomain containing transcription factors Lim3 (Lhx3) and Islet1/2 (Isl1/2) (Lee et al., 2004; Lee and Pfaff, 2003; Mizuguchi et al., 2001; Tsuchida et al., 1994). Downregulation of Olig2 enables Lim3 and Isl1/2 together with the proneural bHLH transcription factor Neurogenin2 (Neurog2) to activate the expression of Hb9, a homeodomain protein and postmitotic motoneuron determinant (Arber et al., 1999; Lee et al., 2005; Ma et al., 2008). In addition, Olig2 cooperates with the homeodomain protein Nkx2.2 to promote oligodendrocyte formation from uncommitted pMN progenitors (Agius et al., 2004; Ligon et al., 2006; Wu et al., 2006). Olig1 mediates gliogenesis redundantly with Olig2 (Lu et al., 2002; Zhou and Anderson, 2002), while Olig3 controls interneuron specification within dorsal neural tube progenitor domains (Ding et al., 2005; Muller et al., 2005; Takebayashi et al., 2002; Zechner et al., 2007). Recent studies uncovered important requirements of Bhlhb4 in retinal bipolar cell maturation (Bramblett et al., 2002; Bramblett et al., 2004), and Bhlhb5 in regulating the specification of retinal amacrine and bipolar cells (Feng et al., 2006), area-specific identity acquisition and axon targeting of cortical postmitotic neurons (Joshi et al., 2008; Ross et al., 2012), as well as differentiation and survival of distinct interneuron subtypes in the spinal cord (Liu et al., 2007; Ross et al., 2010; Skaggs et al., 2011; Xu et al., 2002). In *Drosophila*, genome-wide data base searches identified one single family member, called Olig family (Oli) (Ledent and Vervoort, 2001; Moore et al., 2000; Peyrefitte et al., 2001; Xu et al., 2002), and a recent study described Oli expression in the embryonic ventral nerve cord (VNC) (Zhang et al., 2008). However, despite the central roles of vertebrate Olig family members, the function of their *Drosophila* counterpart has not been investigated.

In *Drosophila*, neurons are derived from stem cell-like neuroblasts (NBs). These divide asymmetrically to generate secondary precursor cells, the ganglion mother cells (GMCs), which divide once to produce two postmitotic neurons and/or glia (Egger et al., 2008; Skeath and Thor, 2003). 15 of 30 embryonic NB lineages give rise to 36 motoneurons in addition to interneurons per abdominal hemisegment (Landgraf and Thor, 2006b; Thor and Thomas, 2002). Zfh1 regulates general motoneuron fate acquisition at the postmitotic level (Garces and Thor, 2006; Layden et al., 2006). The specification of ventrally projecting motoneuron subtypes is mediated by a combinatorial expression of five transcriptional regulators—the fly orthologs of Isl, Lim3, Hb9 and Nkx6, as well as the POU protein Drifter (Dfr; Ventral veinless—FlyBase) (Broihier et al., 2004; Broihier and Skeath, 2002; Certel and Thor, 2004; Odden et al., 2002; Thor et al., 1999; Thor and Thomas, 1997). Many of these determinants are highly conserved, raising the question as to whether Oli functions as part of this genetic network that shapes motoneuron diversity. Although related molecules in vertebrates and invertebrates appear to mediate late aspects of glial function, factors that regulate early steps of gliogenesis and are molecularly and functionally conserved have so far not been identified (Freeman and Doherty, 2006). Olig2 is essential for oligodendrocyte development in vertebrates (Ligon et al., 2006), and a recent study also implicated the *C. elegans* homolog Hlh-17 in regulating gliogenesis (Yoshimura et al., 2008). Thus, Oli is a potential candidate that could control early glial development in *Drosophila*.

Here, we provide insights into the so far unexplored function of the Oli bHLH transcription factor in the *Drosophila* nervous system. Oli is not required in glia; however, taking advantage of the well-defined embryonic motoneuron lineages and axonal projections*,* we demonstrate that *oli* controls trajectory selection and muscle targeting of ventral motoneuron subtypes. Moreover, Oli is expressed in postembryonic lineages, which include glutamatergic leg-innervating motoneurons. Loss-of-function experiments revealed that *oli* is required for larval and adult locomotion. Chick Olig2 can partially rescue these defects in adults, highlighting at least one evolutionarily conserved role of Olig transcription factors in flies and vertebrates.

## Materials and methods

### Molecular biology

*pUAST-oli* and *pUAST-chOlig2* were generated by subcloning *oli* cDNA (*GH17679*; DGRC) into *pUAST* (Brand and Perrimon, 1993) using EcoRI-XbaI restriction sites, and *Olig2* cDNA (kindly provided by J. Briscoe) using Asp718-XbaI sites.

### Drosophila genetics

**Fig. 1:**
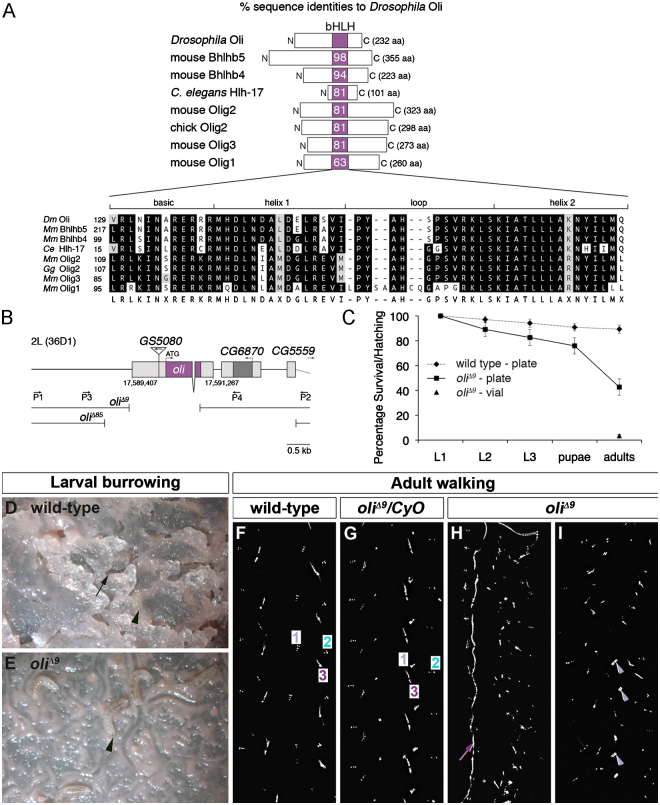
*Drosophila oli* is required for larval and adult locomotion. (A) DNASTAR CLUSTALW and One Pair amino-acid alignments reveal high sequence conservation within the bHLH domain of Olig family members as indicated. *Dm*, *Drosophila melanogaster*; *Mm*, *Mus musculus*; *Ce, Caenorhabditis elegans; Gg, Gallus gallus*. Black boxes highlight identical, grey boxes similar residues. (B) Schematic of *oli* genomic locus and deletions in *oli*^*Δ9*^ and *oli*^*Δ85*^ alleles. (C) Percentage of wild-type and *oli*^*Δ9*^ flies surviving to indicated stages raised on plates, compared to eclosion rate of *oli*^*Δ9*^ adults in crowded vials. Error bars: 95% confidence intervals. (D and E) Wild-type mid 3rd instar larvae (arrowhead, D) dig tunnels (arrow) into fresh plates overnight; *oli*^*Δ9*^ larvae (arrowhead, E) fail to burrow. (F–I) Adult wild-type and heterozygous *oli*^*Δ9*^ flies produce regular traces in leg print assays; 1–3 indicate imprints of the first, second and third left or right legs (F and G). *oli*^*Δ9*^ escapers (H and I) display irregular, shuffling steps and often a dragging leg (arrow). Arrowheads indicate imprints of front legs engaged in grooming. Direction of movement: up.

*oli* mutant alleles were generated by mobilization of the P element *P{GSV2}GS5080* (Kyoto DGRC) inserted into the 5′UTR of *oli* 153 bp upstream of the translation initiation site. Mutant alleles were selected by complementation for lethality with the deficiencies *Df(2L)H20/CyO, Df(2L)Exel9044/CyO* and *Df(2L)Exel7069/CyO* (Bloomington Stock Center). Primers used for break-point analysis (Fig. 1B) were: P1 5′-ATGCGCGAAGTCATTAGGTC; P2 5′-AGTGAATGGCGTTCTGTCTG; P3 5′-TCAGGGTTGAAAAGGAGCGA; P4 5′-AATGCCAGCCGATTTTGCAC.

Progeny of the following lines/crosses, maintained at 25 °C, were analyzed in: (i) *expression and loss-of-function experiments*—(1) wild-type^OreR^, (2) *oli*^*Δ9*^*/CyO*, (3) *oli*^*Δ9*^*/CyO*×*oli*^*Δ85*^*/CyO*, (4) *oli*^*Δ9*^*/CyO*×*Df(2L)H20/CyO,* (5) *oli*^*Δ9*^*/CyO*×*Df(2L)7069/CyO*, (6) *oli*^*Δ9*^*/CyO*×*Df(2L)9044/CyO,* (7) *twi-Gal4; dMef2-Gal4*×*UAS-cd8GFP*, (8) *isl-τ-myc*, (9) *oli*^*Δ9*^*/CyO; isl-τ-myc/TM3* or *TM6B*, (10) *oli*^*Δ9*^*/CyO; isl-τ-myc/TM6B*×*Df(2L)7069/CyO*, (11) *lim3A-τ-myc*, (12) *oli*^*Δ9*^*/CyO; lim3A-τ-myc/TM6B*, (13) *BarH1-Gal4*×*UAS-cd8GFP*, (14) *hb9-Gal4*×*UAS-nGFP*; (ii) *genetic interaction experiments*—(1) *hb9*^*kk30*^*/TM3* or *TM6B*, (2) *hb9*^*kk30*^*/TM3*×*hb9-Gal4/TM3*, (3) *oli*^*Δ9*^*/CyO; hb9*^*kk30*^*/TM3* or *TM6B,* (4) *oli*^*Δ9*^*/CyO; hb9*^*kk30*^*/TM3*×*oli*^*Δ9*^*/CyO; hb9-Gal4/TM3*; (iii) *rescue and gain-of-function experiments*—(1) *elav-Gal4*^*c155*^; *oli*^*Δ9*^*/CyO,* (2) *oli*^*Δ9*^ *sca-Gal4*^*G535−4*^*/CyO,* (3) *oli*^*Δ9*^*/CyO; repo-Gal4/TM3*, (4) *OK371-Gal4 oli*^*Δ9*^*/CyO* each crossed to (5) *w/Y; oli*^*Δ9*^*/CyO; UAS-oli*^*D17−3*^*/TM3*, (6) *elav-Gal4*^*c155*^*; oli*^*Δ9*^*/CyO*×*UAS-oli*^*D3−2*^*/Y; oli*^*Δ9*^*/CyO*, (7) *elav-Gal4*^*c155*^×*UAS-oli*^*D17−3*^, (8) *sca-Gal4*^*G535−4*^×*UAS-oli*^*D17−3*^, (9) *elav-Gal4*^*c155*^*; oli*^*Δ9*^*/CyO*×*w/Y; oli*^*Δ9*^*/CyO; UAS-chOlig2*^*c1−2a*^; for some gain-of-function experiments, wild-type or heterozygous *oli*^*Δ9*^ siblings over-expressing *UAS*-transgenes were tested; (iv) *mosaic analysis with a repressible cell marker* (MARCM; (Lee and Luo, 1999))—(1) *hs-FLP*^*1*^ *elav-Gal4*^*c155*^ *UAS-cd8GFP; tubP-Gal80 FRT40A*×*yw/Y; FRT40A*, (2) *hs-FLP*^*1*^ *tubP-Gal80 FRT19A*×*FRT19A/Y; OK371-Gal4 UAS-cd8GFP/CyO*, (3) *hs-FLP*^*1*^ *UAS-cd8GFP; tubP-Gal80 FRT40A*×*OK371-Gal4 FRT40A*, (4) *hs-FLP*^*1*^ *UAS-cd8GFP; tubP-Gal80 FRT40A*×*OK371-Gal4 oli*^*Δ9*^ *FRT40/Gla Bc*; larvae were heat-shocked for 75 min in a 37 °C water bath 48 h after egg laying; (v) *adult leg innervation analysis*—(1) *OK371-Gal4 FRT40A; UAS-cd8GFP*×*oli*^*Δ9*^ *FRT40A/CyO; UAS-cd8GFP*, (2) *OK371-Gal4 oli*^*Δ9*^ *FRT40A/CyO; UAS-cd8GFP*×*oli*^*Δ9*^ *FRT40A/CyO; UAS-cd8GFP*. Germ-line clones were generated as previously described (Chou and Perrimon, 1996). *Act-GFP* or *Dfd-YFP* labeled *CyO* and *TM6B*, and *Act-GFP TM3* balancers to enable genotyping of embryos.

For rescue crosses (Fig. 9A), the following theoretical percentages of progeny, which are homozygous for *oli*^*Δ9*^ (cross 1) and carry the *UAS*-transgene and Gal4 driver, are expected (crosses 2—7): (1) 33.3%, (2,3,4,6) 16.6%, (5) 8.3%, and (7) 33.3%. To compare similarly populated vials in each experiment, 12 females were crossed to 6 males, and parents were transferred daily into fresh vials for 5 days.

### Immunostaining and antibody generation

Over-night embryo collections were dechorionated and processed in the following ways: (1) entire embryos were fixed in 4% formaldehyde in phosphate-buffered saline (PBS, Invitrogen) for 20 min at room temperature and, subsequent to immunolabeling, imaged as wholemounts or as flat preparations; (2) alternatively, embryos selected by stage and genotype were dissected to generate flat preparations on polylysine-coated slides, essentially as previously described (Landgraf et al., 1997) and fixed in 4% paraformaldehyde in PBS for 20 min at room temperature. The dissected CNS of 3rd instar larvae were fixed in 2% paraformaldehyde in 0.1 M l-lysine containing 0.05 M sodium phosphate buffer for 1 h at room temperature. For immunolabeling, the following primary antibodies were used: mouse anti-BP102 (1:10; Developmental Studies Hybridoma Bank [DSHB]); mouse anti-Dac (1:25; DSHB); rat anti-Drifter (1:2000; Certel and Thor, 2004); mouse anti-Even-skipped (1:10; DSHB); rat anti-Elav (1:25; DSHB); mouse anti-Fasciclin 2 (1:10; DSHB); mouse anti-GFP (1:1000; Roche); rabbit anti-GFP (1:200; Molecular Probes); guinea pig anti-Hb9 (1:500; Broihier and Skeath, 2002); guinea pig anti-Hunchback (1:400; Kosman et al., 1998); goat anti-horseradish peroxidase (HRP)–FITC (1:200; Cappel); mouse mAb24B10 (1:75; DSHB); mouse anti-Miranda (1:50; Ohshiro et al., 2000); mouse anti-Myc mAb9E10 (1:10; DSHB); rabbit anti-Myc (1:250; Santa Cruz Biotechnology); mouse anti-Repo (1:10; DSHB). Secondary immunofluorescent antibodies used were: goat anti-mouse, rabbit, rat and guinea pig F(ab′)_2_ fragments coupled to FITC, Cy5 (1:200), or Cy3 (1:400) (Jackson ImmunoResearch Laboratories), and goat anti-mouse AlexaFluor 488 (1:200; Invitrogen). For TOTO-3 staining (Molecular Probes), samples were incubated for 10 min in a 1:1000 dilution in PBS. Immunofluorescence images were collected using a Zeiss/Bio-Rad Radiance 2100 confocal microscope. For light microscopy, a HRP-conjugated goat anti-mouse secondary antibody (1:200; Bio-Rad) and 3,3′diaminobenzidine as enzymatic substrate were used. Embryo collections, processed to generate flat preparations after fixation, were incubated with both FITC and HRP conjugated secondary antisera; non-fluorescing *oli*^*Δ9*^ embryos were subsequently subjected to the enzymatic reaction. Images were collected using a Zeiss Axioplan 2 equipped with a Jenoptik ProgRes C14 digital camera. Live adult leg preparation were imaged using a Leica SP5 confocal microscope.

To generate a polyclonal antibody against *Drosophila* Oli, two peptides flanking the bHLH domain were employed for rabbit immunizations (Eurogentec): amino acids 68–82 ([C]QPPTDENKPGPSAPE) and 218–232 (LLQGPHNEPPTSSS). For embryonic and larval stainings, antibody dilutions were 1:1000 and 1:2000, respectively.

### Survival and locomotion assays

To monitor survival rates under non-crowded conditions, seven sets of 20–50 wild-type and *oli*^*Δ9*^ 1st instar larvae were placed on yeast paste-supplemented grape-juice agar plates. Larvae were transferred daily to fresh plates to facilitate quantification. As it was not possible to trace numbers of progeny through all developmental stages in crowded vials, the percentage of adult *oli* deficient escapers was obtained from 6 vials. These each contained 12 females crossed to 6 males, and parents were transferred daily into fresh vials for 5 days. Leg print assays were performed as previously described (Maqbool et al., 2006) and imaged under a Leica MZ16F dissecting microscope using a LeicaDC500 digital camera. The contrast of images was enhanced using Adobe Photoshop. The locomotion behavior of 3rd instar larvae on grape juice plates and of adult flies in Falcon petri dishes was filmed using a 3CCD JVC KY-F55B color video camera on a Leica MZ8 dissecting microscope. Movie clips were collected and processed using iMovie software. Details of all protocols are available upon request.

## Results

### oli mutants are semi-lethal and show larval and adult locomotion defects

Our BLASTP searches of *Drosophila* annotated proteins with mouse Olig2 and Bhlhb5 confirmed that the 232 amino-acid long Oli protein is the single most closely related transcription factor to the vertebrate Olig family (Bramblett et al., 2002; Ledent and Vervoort, 2001; Li and Richardson, 2008; Moore et al., 2000; Peyrefitte et al., 2001; Xu et al., 2002). Protein sequence alignments of *Drosophila* Oli, *C. elegans* Hlh-17 and vertebrate Olig family members revealed a high degree of homology within the bHLH domain (63–98%; Fig. 1A).

As basis for functional studies, we generated two independent null alleles, *oli*^*Δ9*^ and *oli*^*Δ85*^, by imprecise P element excision (Fig. 1B). In *oli*^*Δ9*^, sequence analysis determined the breakpoints of a 1.53 kb deletion as 802 bp upstream and 732 bp downstream of the translation start, thus removing most of the *oli* locus, including the bHLH domain-encoding sequence. In *oli*^*Δ85*^, a 4.14 kb deletion extends from 1335 bp upstream of the translation start to 187 bp downstream of the start of *CG5559*, removing *oli*, the adjacent gene *CG6870* and the first exon of *CG5559*.

As 20.1% *oli*^*Δ9*^ homozygous mutant 1st instar larvae hatched from randomly selected eggs (*n*=887, 9 plates) instead of 25% expected, we estimated an embryonic survival rate of 80.4%. When survival during subsequent larval and pupal stages was monitored under non-crowded conditions by maintaining larvae on yeast-supplemented agar plates, we observed a reduced viability of *oli* mutants relative to wild-type (Fig. 1C). 42% of *oli*^*Δ9*^ (*n*=257) compared to 89% of wild-type adult flies (*n*=242) emerged from pupal cases. The eclosion rate of *oli*^*Δ9*^ progeny raised on plates was higher than in populated vials, as on average only 3.4% *oli* deficient adult flies hatched (*oli*^*Δ9*^*/oli*^*Δ9*^, *n*=1135 total progeny, 6 vials). Similarly, on average 2% *oli*^*Δ9*^*/Df(2L)H20* progeny hatched (*n=*636 total progeny) in crosses of balanced *oli*^*Δ9*^ females with *Df(2L)H20* balanced males. Thus, *oli* null mutants are semi-lethal and eclosion rates are affected by a crowded environment.

Wild-type 3rd instar larvae prior to the wandering stage burrow deep tunnels into agar plates (Godoy-Herrera, 1986). By contrast, *oli*^*Δ9*^ larvae of the same age remain largely on the plate surface, indicating that digging behavior is impaired (Fig. 1D and E). Wild-type larvae move by peristaltic crawling, which entails coordinated waves of muscle contractions from posterior to anterior segments (Crisp et al., 2008; Dixit et al., 2008; Suster and Bate, 2002). Video recordings revealed that *oli*^*Δ9*^ mutant larvae crawl with irregular, slower strides, and frequently appear to drag their abdominal segments (Movies S1, S2). All adult *oli*^*Δ9*^ escapers were unable to fly and displayed slow, uncoordinated leg movements, circling and wobbling (Movies S3, S4). In leg print assays (Maqbool et al., 2006), adult wild-type and *oli*^*Δ9*^ heterozygous flies (*n*=18) walking on candle-soot coated glass slides leave behind regular imprints of their three leg pairs, characteristic of an alternating-tripod gait (Fig. 1F and G). By contrast, traces of *oli*^*Δ9*^ escapers are highly irregular because of their uncoordinated leg movements (*n*=10; Fig. 1H and I). Thus, loss of *oli* leads to larval and adult locomotion defects that possibly also affect viability and eclosion rates.

The following is the Supplementary material related to this article Movie S1Movie S2, Movie S3, Movie S4.

Movie S1 . Crawling behavior of wild-type 3rd instar larvae. Wild-type larvae move over substrate by peristaltic crawling in coordinated waves of muscle contractions progressing from posterior to anterior segments.

Movie S2 . Crawling behavior of *oli*^*Δ9*^ 3rd instar larvae. *oli*^*Δ9*^ larvae crawl with less coordinated, slower strides, and appear to drag their abdominal segments.

Movie S3 . Walking behavior of wild-type adult flies. The video clip illustrates the behavior of a fast and smoothly walking adult wild-type fly.

Movie S4 . Walking behavior of *oli*^*Δ9*^ adult flies. Three video clips illustrate the severe locomotion defects observed in adult escapers lacking *oli* function, including slow and uncoordinated leg movements, circling and wobbling.

### Oli is dynamically expressed in the embryonic nervous system

**Fig. S1:**
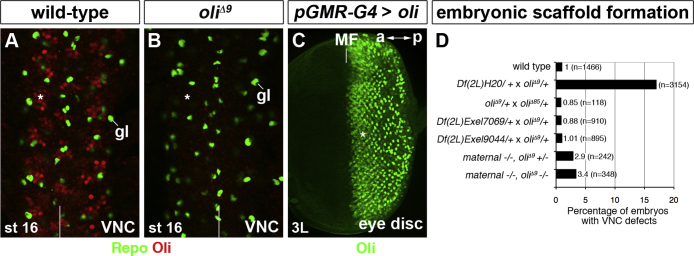
Assessment of Oli antibody specificity; requirement of *oli* in embryonic scaffold formation. (A) Oli (green) is expressed in neuronal nuclei in stage 16 wild-type VNCs. Glial cells (gl) were labeled with Repo (red) serving as landmarks. (B) Expression is absent in oli 9 mutant embryos (asterisks). Speckles are due to unspecific background labeling. White lines: midline. (C) 3^rd^ instar larval eye imaginal discs of flies, in which *pGMR-Gal4* drives ectopic Oli expression using the *UAS-oliD^17-3^* transgene. Oli (green, asterisk) is specifically expressed in cells posterior (p) to the morphogenetic furrow (MF), including differentiated photoreceptor neurons. a, anterior. (D) Percentages of embryos displaying axon scaffold formation defects were determined for crosses, in which flies heterozygous for *oli^Δ9^* and a wild-type chromosome were crossed to heterozygous flies carrying *oli^Δ85^* or deficiency containing chromosomes over wild-type chromosomes. Wholemount embryos were labeled with BP102. Crosses, which contained embryos transheterozygous for *oli^Δ9^* and *oli^Δ85^*, or *oli^Δ9^* and the deficiencies *Df(2L)Exel7069 and Df(2L)Exel9044* did not show significant proportions of specimen with defects. Crosses containing *oli^Δ9^*/*Df(2L)H20* embryos showed a high percentage of embryos with severe defects (17%, n=3154) compared to controls (1%, n=1466). Defects were solely observed in embryos transheterozygous for *oli* and *Df(2L)H20*, but not the other deficiencies; they are likely due to yet to be identified gene(s) within the deficiency acting as enhancer(s). Removal of a possible maternal contribution by generating germline clones did not reveal any significant role in axon scaffold formation; only 2.9% of embryos lacking maternal *oli* (*oli^Δ9^* +/−, n=348) showed defects compared to 3.4% of embryos lacking zygotic and maternal *oli* (*oli^Δ9^* −/−, n=242).

**Fig. 2:**
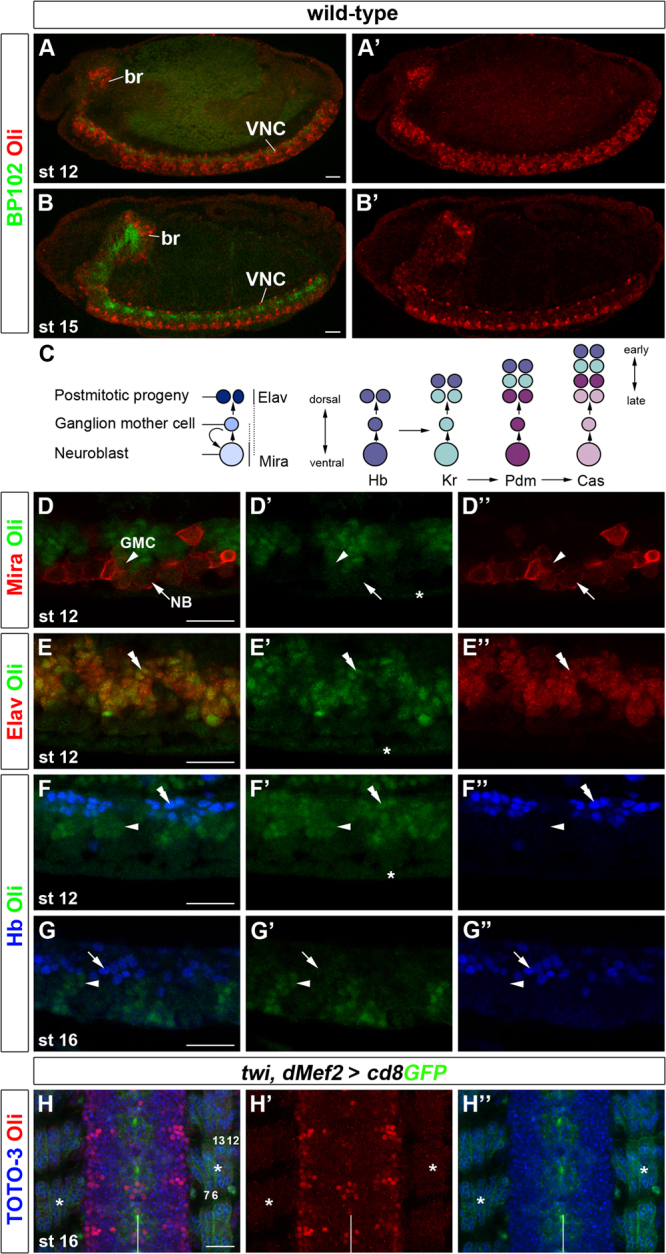
Oli is dynamically expressed in neuronal progeny. (A–B') Lateral views of wholemount stage 12 and 15 embryos showing that Oli (red) is specifically expressed in the brain (br) and ventral nerve cord (VNC). Axonal tracts were labeled with BP102 antiserum (green). (C) Schematic representation of embryonic neurogenesis in *Drosophila*. NB, neuroblast; GMC, ganglion mother cell. Ventrally located NBs sequentially express members of the temporal transcription factor series: Hunchback (Hb), Kruppel (Kr), Pou-homeodomain proteins 1/2 (Pdm) and Castor (Cas). GMCs and to some extent their progeny maintain the expression of the temporal factor present in the NB at the time of their birth. Early-born progeny are shifted dorsally by later-born neurons. (D–F") Stage 12. Oli protein (green) was detected in some GMCs (arrowheads), but not in NBs (arrows) labeled with Mira (red) (D–D"). It is widely expressed in postmitotic Elav-positive neurons (red, double arrowheads) (E–E"). Expression is detected in Hb-positive (blue, double arrowheads) and Hb-negative neurons (arrowheads) (F–F"). Asterisks in D', E', F' indicate background labeling in epidermis. (G–G") Stage 16. Oli is downregulated in Hb-positive neurons (arrows), but is present in some ventrally located, later-born neurons (arrowheads). (H–H") Stage 16. Oli (red) is detected in neuron subtypes in the VNC, but not in muscles 7, 6, 13 and 12 (asterisks) visualized with *twi-Gal4; dMef2-Gal4* driving *UAS-cd8GFP* expression (green). Nuclei are labeled with TOTO-3 (blue). (A–G") Anterior: left. (H–H") Anterior: up; vertical lines: midline. Scale bars 20 μm.

The locomotion defects are likely due to a requirement of *oli* in the nervous system. To obtain first clues regarding the role of *oli*, we examined its expression using an Oli-specific polyclonal antibody (Fig. S1A–C). Consistent with in situ hybridization stainings (Zhang et al., 2008), Oli protein was specifically detected in the embryonic nervous system (Fig. 2A–B'). Co-staining with Miranda (Mira; (Ikeshima-Kataoka et al., 1997)) showed that Oli is not expressed at detectable levels in NBs, but is present in some GMCs (Fig. 2C–D"). Elav (Embryonic lethal, abnormal vision) labels primarily differentiated neurons, and transiently NBs and glia at early stages (Berger et al., 2007; Robinow and White, 1991). Hunchback (Hb), a member of the temporal transcription factor series, is expressed in early-born NBs, GMCs and postmitotic progeny (Brody and Odenwald, 2000; Grosskortenhaus et al., 2005; Isshiki et al., 2001; Kambadur et al., 1998). Staining with these markers (Fig. 2E–G") revealed wide Oli expression in differentiated neurons during intermediate embryonic stages (10–13), including those that are Hb-positive and, thus, early-born. While expression is downregulated in many neurons during subsequent stages (14–17), Oli is detected in subsets of neurons, some of which are later-born, as they are Hb-negative and located more ventrally within the VNC. Examining 10 embryos (*n*=80 hemisegments) at stage 16, we estimated that each hemisegment contains approximately 37.3 Oli-positive neurons (±4.58, 95% confidence interval). The variability is likely due to the dynamic nature of the expression pattern. Oli expression was not detected in embryonic muscles (Fig. 2H–H"). Hence, the expression in postmitotic neurons suggests a role for *oli* in controlling neuronal development.

### oli is not required for glial development

**Fig. 3:**
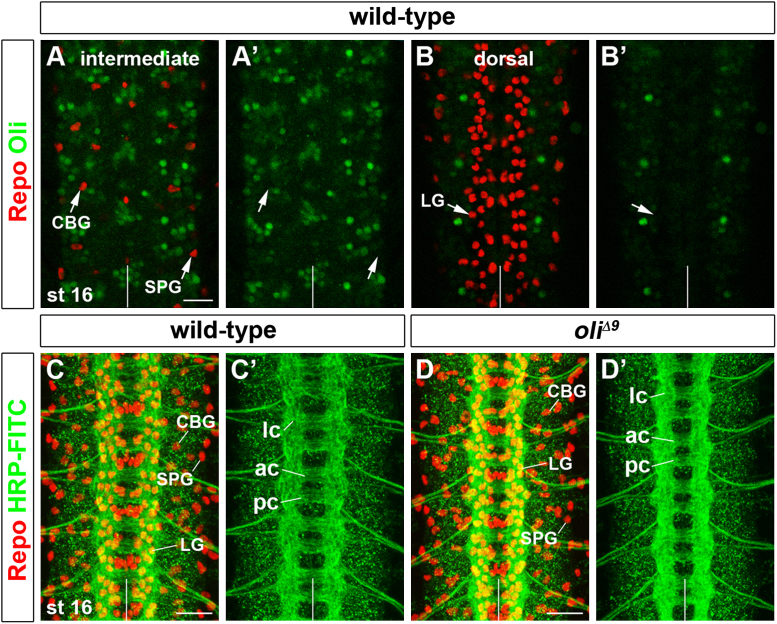
Oli is not expressed in glial progeny and not required for gliogenesis in the embryonic VNC. (A–D') Stage 16. Repo-positive (red) subperineurial (SPG), cell body (CBG) and longitudinal (LG) glia (arrows) located in intermediate and dorsal focal planes do not express Oli (green) (A–B'). The number and distribution of SPG, CBG, and LG Repo-positive glia (red) is similar in wild-type and *oli*^*Δ9*^; also axonal scaffold organization, consisting of longitudinal connectives (lc), and anterior and posterior commissures (ac, pc) visualized with HRP-FITC (green), is normal (C–D'). Anterior: up; vertical lines: midline. Scale bars 20 μm.

As vertebrate Olig2 is required for oligodendrocyte formation, we explored a possible function of *Drosophila oli* in glia. Unexpectedly, co-labeling with Oli and the homeodomain protein Reversed polarity (Repo), a broad glial marker (Halter et al., 1995; Xiong et al., 1994), did not reveal any obvious overlap of expression in the CNS of stage 10–16 embryos (*n*=41; Fig. 3A–B'). Moreover, staining with Repo and anti-HRP to visualize glia and axonal tracts did not uncover defects in general scaffold formation in *oli*^*Δ9*^ stage 16 embryos (Fig. 3C–D'; see also S1D). In line with the recent survey of embryonic VNC glia (Beckervordersandforth et al., 2008), we detected about 25 glial cells per abdominal hemisegment at characteristic positions in wild-type (24.88 cells/hemisegment; *n*=113; Fig. 3C and C'). Neither the distribution nor the number of glia were altered in *oli*^*Δ9*^ embryos (24.53 cells/hemisegment; *n*=118; Fig. 3D and D'), suggesting that *oli* is not essential for embryonic gliogenesis.

### Oli is expressed in ISNb, TN and SNa motoneurons

**Fig. 4:**
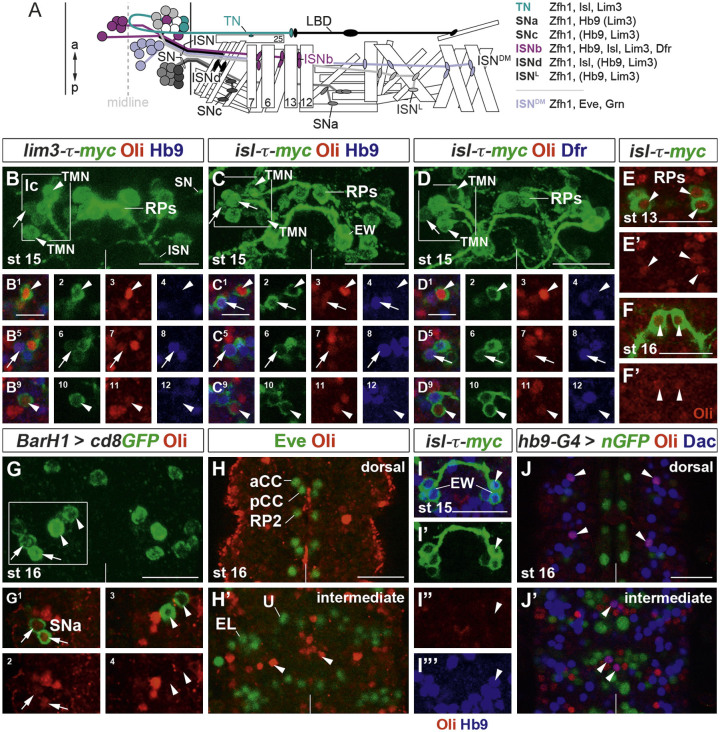
Oli is expressed in ISNb, TN and SN motoneurons in the embryonic VNC. (A) Schematic representation of ISN, SN and TN motoneuron subtypes, their axonal trajectories, muscle innervation patterns and defining combination of transcription factors (modified from Landgraf and Thor, 2006a). a: anterior; LBD, lateral bipolar dendrite neuron; p: posterior; B, C, D, H represent projections of confocal stacks; panels below show single optical sections. (B–D^12^) Stage 15. Oli (red), Hb9, Dfr (blue), *lim3-τ-myc* and *isl-τ-myc* transgenes (green) are co-expressed in 1–2 ISNb motoneurons (arrows) within the lateral cluster (lc, white boxes). Two Hb9- and Dfr-negative transverse nerve motoneurons (TMNs, arrowheads) express different levels of Oli. (E–F') *isl-τ-myc*-positive RP neurons (arrowheads) express Oli at stage 13; levels decrease at stage 16. (G–G^4^) Lateral (arrows), but not medial SNa motoneurons (arrowheads) labeled with *BarH1-Gal4, UAS-cd8GFP* express Oli at stage 16. (H and H') Dorsal aCC, pCC and RP2 neurons, and U and Eve lateral (EL) neurons at intermediate levels express Eve (green), but not Oli (arrowheads) at stage 16. (I–I"') Serotonergic *isl-τ-myc-*, Hb9-positive EW interneurons (arrowheads) do not express Oli at stage 15. (J and J') A subset of dorsally and medially located Dac-positive interneurons (blue) co-express Oli (arrowheads) at stage 16. Hb9 expressing neurons were visualized using *hb9-Gal4* and *UAS-nGFP* (green). Vertical lines: midline. Scale bars 20 μm (10 μm in B^1^, C^1^ and D^1^).

Because vertebrate Olig2 is central for somatic motoneuron development and *oli* mutant larvae displayed locomotion defects, we examined next whether Oli-positive subpopulations included motoneurons. In each hemisegment, axons of motoneuron subtypes exit the CNS via the intersegmental (ISN), segmental (SN) and transverse (TN) nerves in a defined pattern (Fig. 4A; (Landgraf and Thor, 2006a)). Motoneurons within the ISNb, ISNd and ISN^L^ sub-branches innervate ventral and lateral internal muscles, while SNa and SNc motoneurons extend to ventral external muscles. Two motoneurons (TMNs) exit the CNS via the TN at the dorsal midline and project distally along segment boundaries. Finally, ISN^DM^ motoneurons connect with dorsal internal muscles. Ventrally and laterally projecting motoneurons are characterized by the combinatorial expression of Lim3, Isl, Hb9 and Dfr (Broihier and Skeath, 2002; Certel and Thor, 2004; Odden et al., 2002; Thor et al., 1999; Thor and Thomas, 1997), while dorsally projecting motoneurons express Even-skipped (Eve) and Grain (Garces and Thor, 2006; Landgraf et al., 1999).

Co-labeling of Oli with ventral motoneuron determinants (Fig. 4B–D^12^) revealed that at stage 15, 1–2 lateral Oli expressing neurons are also positive for *lim3-τ-myc*, *isletH-τ-myc (isl-τ-myc)*, Hb9 and Dfr and, thus, correspond to ISNb motoneurons. Oli expression in these neurons is frequently downregulated from stage 16 onwards. Two more lateral Oli-positive neurons can be identified as TMNs, since they are Hb9- and Dfr-negative and their axons exit via the TN. While medial RP1/3/4/5 neurons express Oli at stage 13, levels subsequently decrease (Fig. 4E–F'). Moreover, Oli was detected in *BarH1-Gal4*-positive SNa motoneuron subtypes (Garces et al., 2006) (Fig. 4G–G^4^). Finally, co-labeling with Eve did not reveal any apparent overlap with Oli in the dorsally projecting aCC, RP2 and U1–5 motoneurons and in pCC and Eve lateral (EL) interneurons (Broihier and Skeath, 2002; Landgraf et al., 1999) at stage 16 (Fig. 4H and H').

To explore further as to whether Oli-positive neuronal subtypes in the embryonic CNS include interneurons, we focused on the well-characterized *isl-τ-myc*-, Hb9-positive serotonergic EW interneurons (Odden et al., 2002), but did not detect any expression (Fig. 4I–I"'). However, Oli was found in some dorsally and medially located interneurons defined by expression of the transcription factor Dachshund (Dac) (Miguel-Aliaga et al., 2004) (Fig. 4J and J'). Together, this indicates that Oli, albeit not exclusively, is expressed in a subset of ventrally projecting motoneurons.

### oli mutant embryos display motoneuron projection defects

**Fig. S2:**
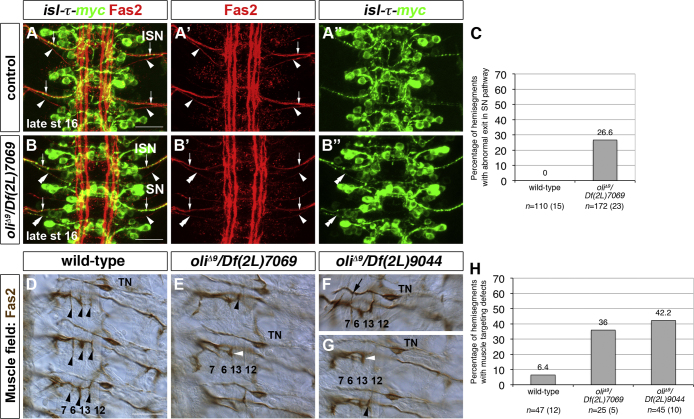
SN exit and muscle innervation defects in *oli* deficient embryos. (A-B“) Late stage 16. In wild-type, *isl-τ-myc*-positive axons (green) project through the ISN (arrow) but not the SN (C-C“, arrowhead) labelled with Fas2 (red). In *oli^Δ9^*, some *isl-τ-myc*-positive motoneurons abnormally extend along the SN (D-D“, double-arrowheads). Anterior: up. (C) Quantification of projection defects. (D-G) Stage 17. In wild-type (D), Fas2-positive ISNb motoneurons innervate the clefts of muscles 6, 7, 12 and 13 (black arrowheads). In *oli^Δ9^* over *Df(2L)7069 (E) and Df(2L)9044* (F, G), some motoneurons axons fail to innervate muscles 13/12 (white arrowheads), or ISNb and TN axons make abnormal contacts (black arrows). (D-G) Anterior: up; VNC: left. (H) Quantification of projection defects. Scale bars 20 μm.

**Fig. 5:**
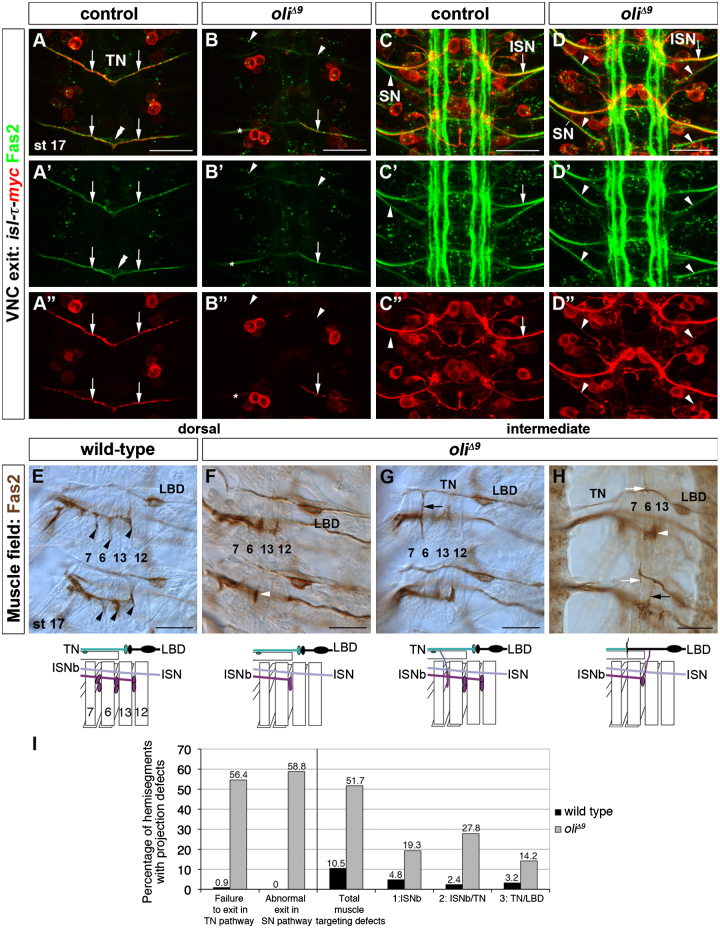
*oli* mutant embryos display VNC exit and muscle innervation defects. (A–D") Stage 17. In wild-type, TN branches labeled with Fas2 (green) and *isl-τ-myc* (red) exit from the dorsal VNC surface (A–A", arrows). *isl-τ-myc*-positive axons project through the ISN (arrow) but not the SN (C–C", arrowhead). Double arrowheads, exit glia. In *oli*^*Δ9*^, some *isl-τ-myc*-positive motoneurons fail to exit via the TN (B–B", arrowheads), and abnormally extend along the SN (D–D", arrowheads). Asterisks in B', B" indicate ISN branches visible in this projection of three optical sections. Anterior: up; vertical lines: midline. (E–H) Stage 17. In wild-type (E), Fas2-positive ISNb motoneurons innervate the clefts of muscles 6, 7, 12 and 13 (black arrowheads). In *oli*^*Δ9*^ (F–H), motoneurons axons fail to innervate muscles 13/12 (white arrowheads, phenotype 1: ISNb), ISNb and TN axons make abnormal contacts (black arrows, phenotype 2: ISNb/TN), or processes from the TN/LBD fascicle project abnormally towards ventral muscles (white arrows, phenotype 3: TN/LBD). (E–H) Anterior: up; VNC: left. (I) Quantification of projection defects. Scale bars 20 μm.

**Fig. 6:**
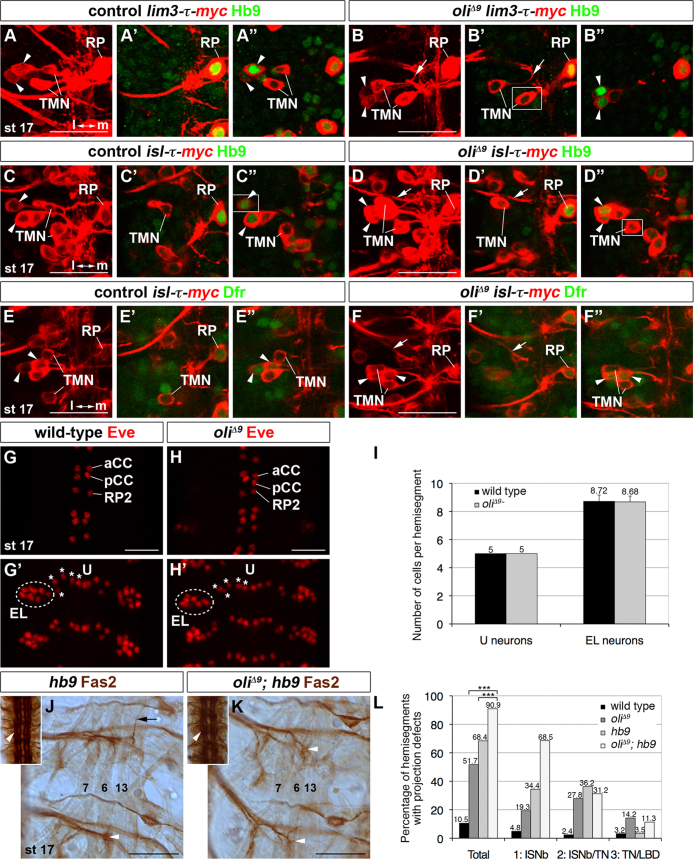
*oli* and *hb9* synergistically regulate ISNb axon targeting. (A–F") Stage 17. Expression of *lim3-τ-myc, isl-τ-myc* (red), Hb9 and Dfr (green) appears unaffected in *oli*^*Δ9*^. A, B, C, D, E and F: projections; adjacent panels: single optical sections. Cells in white boxes originate from an immediately adjacent section. TMNs express *lim3-τ-myc* and *isl-τ-myc*, but not Hb9 and Dfr, and RP neurons express all four markers in wild-type (A–A", C–C", E–E") and *oli*^*Δ9*^ (B–B", D–D", F–F"). *lim3-* and *isl-*positive axons, projecting incorrectly into the SN (arrows) do not express Hb9 or Dfr in *oli*^*Δ9*^. Hb9 and Dfr are normally expressed in two lateral cells (arrowheads). Anterior: up; VNC: left. l, lateral; m, medial. (G–H') Stage 17. Eve expression (red) in aCC, pCC, RP2, U (asterisks) and EL (circled) neurons is similar in wild-type (G and G') and *oli*^*Δ9*^ (H and H'). Anterior: up. (I) Quantification of U and EL neurons. Error bars: 95% confidence intervals. (J, K and insets) Stage 17. In *hb9*, Fas2-positive ISNb motoneurons fail to innervate the cleft between muscles 12/13 (white arrowhead) or abnormally contact the TN (black arrow) (J). In *oli*^*Δ9*^*; hb9*, defects are enhanced (white arrowheads) (K). Insets show largely normally fasciculated Fas2-positive bundles (arrowheads) in the abdominal CNS of samples shown in J and K. The outer fascicles appear discontinuous likely because of the age of embryos. Anterior: up; VNC: left. (L) Quantification of muscle innervation defects. *p*=2.7×10^−7^ (*oli* and *oli; hb9*) and *p*=0.0013 (*hb9* and *oli; hb9*) χ^2^-test. Asterisks indicate statistically significant differences. Scale bars 20 μm.

The expression of Oli suggests a possible role in regulating ventral motoneuron subtype development. We therefore assessed the axonal trajectories of motoneurons within the CNS and the muscle target field. In wild-type, *isl-τ-myc*-positive motoneuron axons leave the VNC via the ISN and TN, but not SN branches, that were co-labeled with Fasciclin 2 (Fas2) (Thor and Thomas, 1997; Van Vactor et al., 1993). By contrast in *oli*^*Δ9*^ mutants, *isl-τ-myc*-labeled axons frequently failed to exit via the TN (54.6%, *n*=119 hemisegments *oli*^*Δ9*^; 0.9%, *n*=110 wild-type). Strikingly, *isl-τ-myc*-positive motoneurons ectopically extended axons through the SN branch in *oli*^*Δ9*^ VNCs (58.8% hemisegments compared to 0% in wild-type; Fig. 5B, B", D, D", I). A similar phenotype was also observed in a *oli*^*Δ9*^*/Df(2L)7069* genetic background (Fig. S2A–C). These neurons expressed neither Hb9 nor Dfr (*n*=10 stage 16/17 embryos; Fig. 6D–D" and F–F"), suggesting that their projections corresponded to TMN axons abnormally extending along the SN.

Trajectory formation in the muscle field relies on a tightly regulated sequence of fasciculation and defasciculation events, enabling motoneurons to leave their nerve branches and innervate specific target muscles. In wild-type, ISNb motoneurons innervate the clefts of muscles 6, 7, 12 and 13. One TMN axon contacts the ventral muscle 25, whereas the other connects with the ventral process of the lateral bipolar dendrite (LBD) neuron, a specialized peripheral neuron contacting the dorsal visceral alary muscles of the heart and aorta (Gorczyca et al., 1994; Thor and Thomas, 1997). In *oli*^*Δ9*^ embryos, ISNb and TN motoneurons exhibited conspicuous muscle targeting defects (51.7%, *n*=176 hemisegments *oli*^*Δ9*^; 10.5%, *n*=124 wild-type) (Fig. 5E–I, Table S1): (i) ISNb motoneuron axons did not correctly defasciculate from each other, leading to the stalling of thicker axon bundles between muscles 6 and 13 and failure to innervate the cleft between muscles 12/13 (phenotype 1: 19.3% hemisegments *oli*^*Δ9*^; 4.8% wild-type); (ii) ISNb or TN axons extended processes that formed abnormal contacts between them (phenotype 2: 27.8% hemisegments *oli*^*Δ9*^; 2.4% wild-type); and (iii) LBD/TN neurons extended ectopic processes onto ventral muscles (phenotype 3: 14.2% hemisegments *oli*^*Δ9*^; 3.2% wild-type). Qualitatively similar phenotypes were also detected in *oli*^*Δ9*^*/Df(2L)7069* and *oli*^*Δ9*^*/Df(2L)9044* mutant embryos (Fig. S2D–H). Thus, in addition to the known determinants *Nkx6*, *isl*, *lim3*, *hb9* and *dfr*, we have identified *oli* as a regulator of ventral motoneuron subtype development, that mediates correct axon pathfinding of TMNs and muscle targeting of ISNb and TN motoneurons.

### oli and hb9 coordinately regulate ISNb axon targeting

**Fig. S3:**
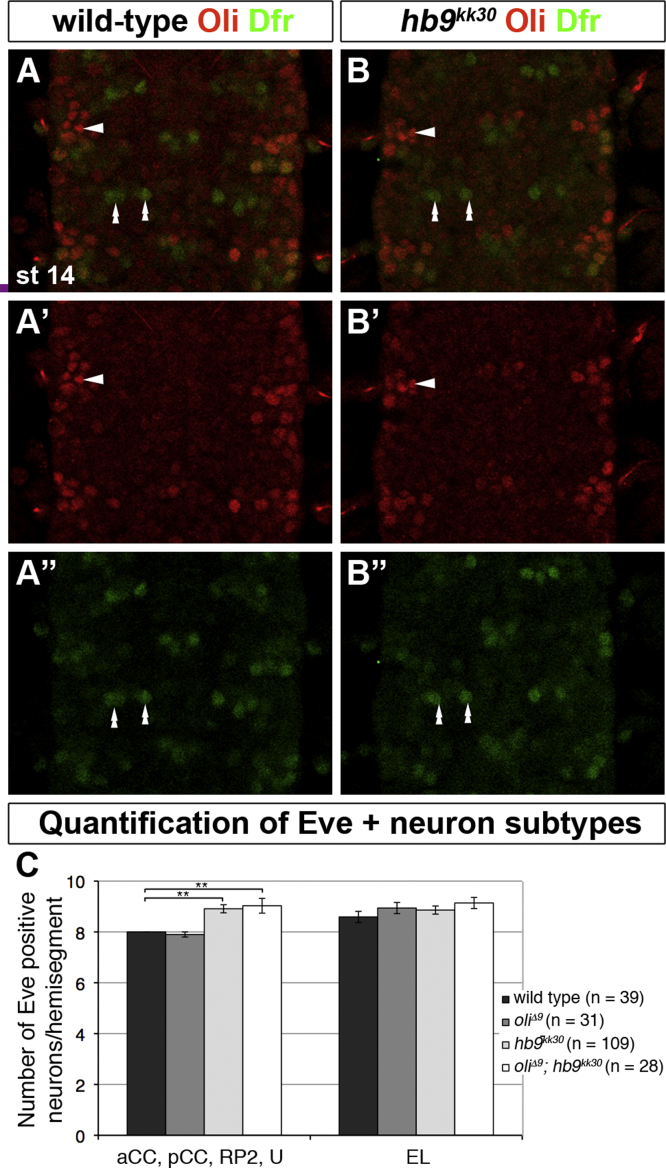
General expression of Oli and Dfr is not affected in hb9 mutant embryos. Confocal images taken at comparable levels show Oli expression in lateral clusters (red, arrowheads), as well as Dfr expression in individual cells (green, double arrowheads) in similar patterns in wild-type (A-A”) and *hb9^kk30^* stage 14 embryos (B-B”). Anterior: up. (C) Quantification of Eve expressing neurons per hemisegment of stage 16 embryos. The number of Eve-positive aCC, pCC, RP2 and U neurons is significantly increased in *hb9^kk30^* compared to wild-type and *oli^Δ9^* embryos, but does not increase further in *oli^Δ9^*; *hb9^kk30^* double mutants. Error bars: 95% confidence intervals. Asterisks indicate statistically significant differences using student t-test analysis.

*isl*, *lim3*, *hb9* and *dfr*—at least for the so far tested combinations—are not transcriptional targets of each other in ventral motoneurons (Broihier and Skeath, 2002; Certel and Thor, 2004). Similarly, when we examined their expression in wild-type and *oli*^*Δ9*^ embryos at stage 17, we did not detect any apparent defects in ISNb and TN motoneurons (Fig. 6A–F"). Also homozygous mutant *hb9*^*KK30*^ embryos at stage 14 did not show altered Oli expression at this resolution (Fig. S3A–B").

Vertebrate Olig2 mediates progenitor domain formation through cross-repressive transcriptional interactions (Briscoe and Novitch, 2008), and also Eve and Hb9 mutually repress their expression in distinct neuronal subsets (Broihier and Skeath, 2002; Odden et al., 2002). We therefore examined Eve expression in wild-type and *oli*^*Δ9*^ stage 17 embryos, and observed that the number and positions of Eve-positive neurons were unaltered with 3 aCC, pCC and RP2, 5 U and 8–9 EL neurons detectable per hemisegment (8.72 EL neurons, *n*=22 hemisegments wild-type; 8.68 EL neurons, *n*=34 *oli*^*Δ9*^; Fig. 6G–I). Hence, Oli neither acts upstream of *isl*, *lim3*, *hb9* and *dfr* in the examined motoneuron subtypes, nor does its own general expression depend on *hb9*. Moreover, *oli* does not influence the choice of ventral versus dorsal motoneuron fates. It may thus act in concert with the other ventral determinants to shape TN and ISNb motoneuron pathfinding and targeting.

To test this hypothesis, we focused on a potential genetic interaction between *oli* and *hb9* (Fig. 6J–L, Table S1) in regulating targeting to muscles. As both *hb9*^*KK30*^ homozygous and *hb9*^*KK30*^*/hb9-Gal4* transheterozygous embryos displayed qualitatively similar motoneuron projection defects, both genotypes were analyzed. Removal of *oli* and *hb9* did not lead to an increase of Eve-positive neurons compared to *hb9* single mutants (Fig. S3C). However, the penetrance of ventral motoneuron projection defects strongly increased in double mutants (90.9% of *n*=54 hemisegments *oli*^*Δ9*^*; hb9*) compared to single mutants (51.7% of *n*=176 hemisegments *oli*^*Δ9*^; 68.4% of *n*=166 *hb9*). Targeting of ISNb motoneuron axons was most strongly affected by the loss of both determinants (phenotype 1): While ISNb axons failed to properly defasciculate and innervate the cleft between muscles 12/13 in 19.3% (*oli*^*Δ9*^) and 34.4% *(hb9*) of hemisegments, the penetrance of this phenotype was enhanced to 68.5% in *oli*^*Δ9*^*; hb9* double mutants. This suggests that *oli* and *hb9* synergistically regulate targeting of at least one motoneuron subtype, the ISNb neurons, in which they are co-expressed.

### Timing and levels of oli expression are critical for motoneuron axon targeting

**Fig. 7:**
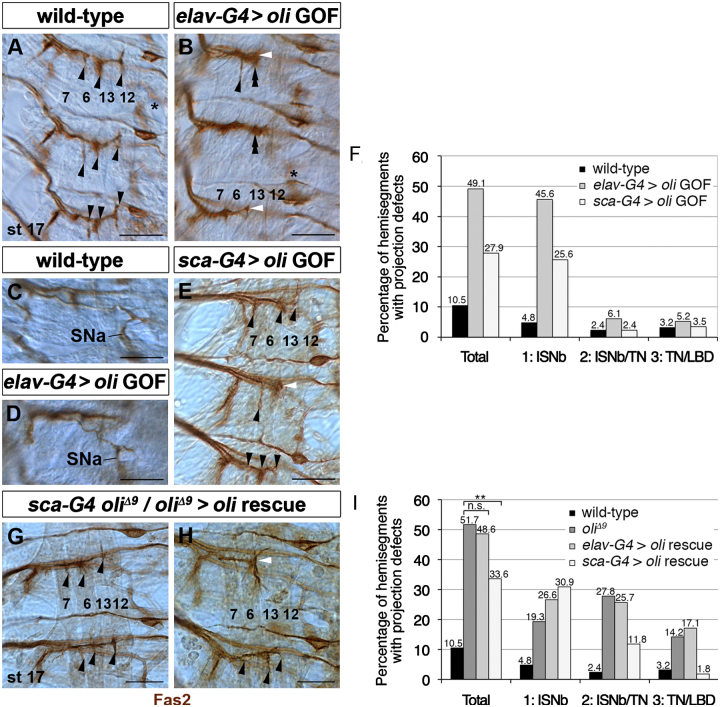
Timing and levels of Oli expression are critical for motoneuron axon targeting. (A–E, G–H) Stage 17. In wild-type (A), Fas2-positive ISNb motoneurons innervate the clefts of muscles 6, 7, 12 and 13 (black arrowheads). (B) In *elav-Gal4*^*c155*^*/+* or *Y; UAS-oli/+* embryos, ISNb motoneurons form thick bundles extending short processes (double arrowheads) or fail to innervate their correct target muscles 13/12 (white arrowheads). Panels C and D show the SNa projections of segments labeled with asterisks in panels A and B. (E) In *sca-Gal4/+; UAS-oli/+* embryos, ISNb motoneurons fail to innervate their correct target muscles 13/12 (white arrowheads). (F) Quantification of muscle innervation defects. (G and H) In *oli*^*Δ9*^*sca-Gal4*/ *oli*^*Δ9*^*; UAS-oli/+* embryos, motoneuron projection defects are partially rescued. Some ISNb motoneurons project normally to their target muscles (black arrowheads); others fail to innervate the clefts of muscles 12/13 (white arrowheads). (I) Quantification of muscle innervation defects in wild-type, *oli*^*Δ9*^, *elav-Gal4*^*c155*^*/+* or *Y*; *oli*^*Δ9*^*; UAS-oli/+* and *oli*^*Δ9*^*sca-Gal4*/*oli*^*Δ9*^*; UAS-oli/+* embryos. *p*=0.611 (*oli* and *elav-Gal4* rescue) and *p*=0.002 (*oli* and *sca-Gal4* rescue) χ^2^-test. Asterisks indicate statistically significant differences. Scale bars 20 μm.

Because of the transient and dynamic expression in motoneuron subtypes throughout embryonic development, we next tested whether altering Oli expression in the VNC may affect muscle innervation. We generated transgenic lines for over-expression of full-length Oli (*UAS-oli*^*D17−3*^, *UAS-oli*^*D3−2*^) and confirmed their efficiency in eye imaginal discs (Fig. S1C; data not shown). When *elav-Gal4*^*c155*^ was used to over-express *UAS-oli*^*D17−3*^ strongly in postmitotic neurons, including ISNb motoneurons, which normally downregulate Oli during late embryonic development, many hemisegments (49.1%, *n*=114) displayed motoneuron projection defects compared to controls (Fig. 7A, B, F, Table S1). ISNb axons frequently failed to defasciculate and to correctly innervate the clefts between target muscles (45.6%). As for instance, SNa axons are clearly discernible (Fig. 7C and D), fasciculation defects are likely not caused by misrouting of other motoneuron subtypes. Expression with *sca-Gal4*, a driver primarily active in NBs, GMCs and to some extent their progeny (Klaes et al., 1994), caused less severe defects (27.9%, *n*=86; Fig. 7E and F, Table S1). Consequently, expression with *elav-Gal4*^*c155*^ in an *oli*^*Δ9*^ mutant background did not rescue, as 48.6% of hemisegments (*n*=105) displayed aberrant projections (Fig. 7I). However, expression with *sca-Gal4* led to a partial rescue, as the penetrance of targeting defects decreased to 33.6% (*n*=110 hemisegments) compared to 51.7% in *oli*^*Δ9*^. While ISNb motoneuron targeting defects remained abundant (phenotype 1: 30.9%), the frequency of aberrant contacts between ISNb and TN motoneurons decreased to 11.8% (phenotype 2; Fig. 7G–I, Table S1). These findings suggest that the tight regulation of timing and/or levels of Oli activity, especially in ISNb motoneurons, is essential for correct trajectory formation.

### Oli is expressed in postembryonic glutamatergic lineages

In a second neurogenic phase during larval and early pupal development, NBs produce adult-specific neurons, and indeed the majority of leg-innervating motoneurons are born during larval life (Brierley et al., 2009; Brierley et al., 2012). Locomotion behavior thus depends on the coordinated activity of dedicated neuronal networks, which arise during embryonic and postembryonic development. We therefore asked, whether *oli* may have an additional postembryonic requirement that contributes to the adult walking defects.

**Fig. S4:**
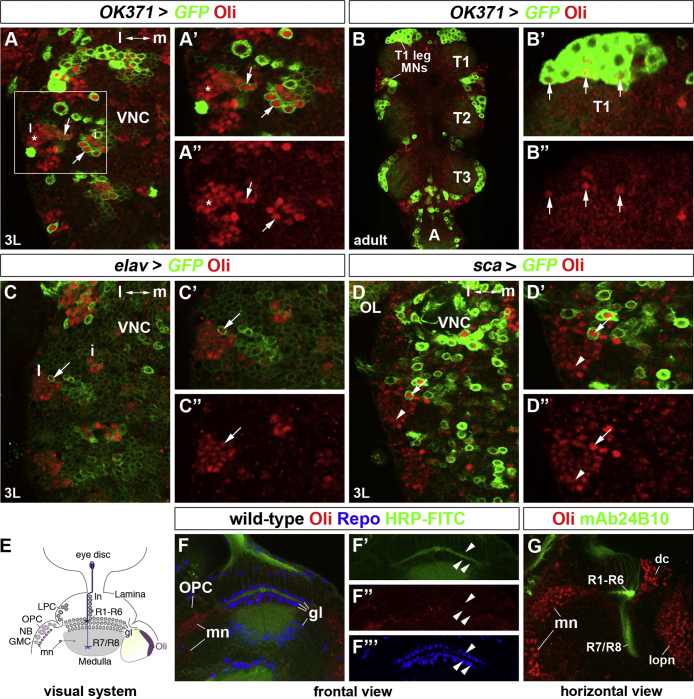
Postembryonic and adult expression of Oli. (A-A”) In 3^rd^ instar larval (3L) VNCs, Oli (red) is expressed in many neurons (arrows) labeled with *OK371-Gal4* and *UAS-cd8GFP* within the lateral (l) and intermediate (i) domains. m, medial. Asterisks indicate Oli-positive, GFP-negative neurons. (B-B”) In thoracic neuromeres of adult VNCs, Oli expression is maintained in some neurons that are labeled with *OK371-Gal4* and *UAS-cd8GFP*. These are located within clusters located anteriorly of the T1 neuropil, containing leg motoneurons (arrows). B’ and B” show higher magnifications of the left anterior T1 neuromere cluster. (C-C”) *elav-Gal4^c155^* drives expression of GFP in all postmitotic neurons that are positive for Oli (red, arrows). l, lateral; i, intermediate; m, medial domains. (D) *sca-Gal4* expression is restricted to some postmitotic progeny and only partially overlaps with Oli expression. Arrows indicate Oli- and GFP-positive neurons; arrowheads indicate Oli-positive and GFP-negative neurons. (E) Schematic representation of a 3^rd^ instar larval visual system. Photoreceptor neurons (R-cells, R1-R8) in the eye imaginal disc extend axons into the optic lobe. R1-R6 axons terminate in the first optic ganglion, the lamina, between two rows of lamina glia. R7 and R8 axons terminate in the second optic ganglion, the medulla. A third row of medulla glia separates the lamina and medulla. Neuroepithelial cells within the outer proliferation center (OPC) produce laterally lamina precursor cells (LPCs), which in turn divide to generate lamina neurons (ln). Neuroepithelial cells generate medially NBs, which divide and give rise to GMCs and medulla neurons (mn). These are spatially organized into rows, whose position reflects their order of birth. Newly-born neurons are located closest to the OPC. (F-F”’) Oli (red) is not expressed in Repo-positive glia in the optic lobe (blue, arrowheads). The development of lamina glia is not affected in the absence of *oli* (IS, unpublished observations). (F and G) Oli is detected in young medulla neurons (mn) beneath the OPC and in the lateral medulla cell body cortex. Oli is expressed in distal cells (dc) and lobula plate neurons (lopn).

**Fig. 8:**
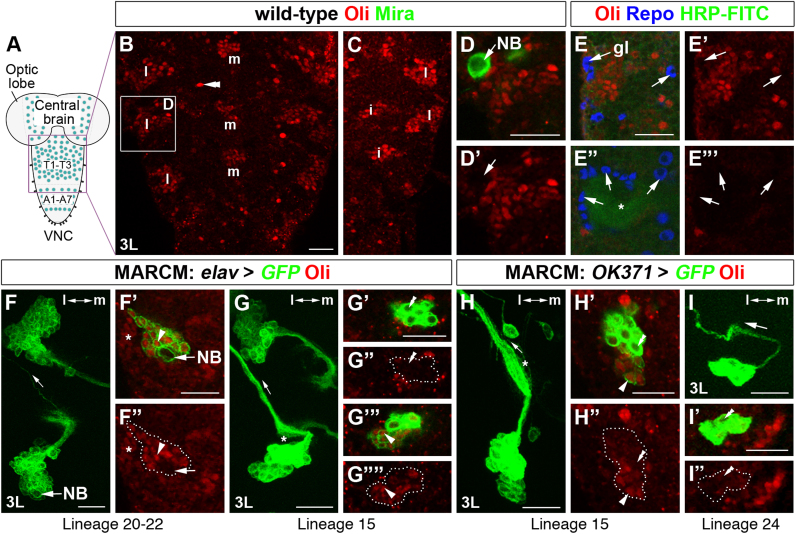
Oli is expressed in postembryonic leg-innervating neuron lineages. (A) Schematic representation of a 3rd instar larval CNS; purple box indicates the thoracic segment area shown in B; green circles, neuroblasts. (B and C) Oli (red) is expressed in individual cells (double arrowheads), and within lateral (l), intermediate (i) and medial (m) domains shown in different focal planes of two samples. Oli is neither expressed in postembryonic Mira-positive neuroblasts (green, NB, arrows, D, D') nor in Repo-positive cell body or neuropil-associated glia (blue, gl, arrows, E–E"'). Asterisk, thoracic neuropil labeled with HRP-FITC (green). (F–G"") *elav-Gal4*^*c155*^ MARCM: *hs-FLP*^*1*^*elav-Gal4*^*c155*^*UAS-cd8GFP/+* or *Y; tubP-Gal80 FRT40A/FRT40A*. (H–H") *OK371-Gal4* MARCM: *hs-FLP*^*1*^*tubP-Gal80 FRT19A/FRT19A; OK371-Gal4 UAS-cd8GFP/+.* (I–I") *OK371-Gal4* MARCM: *hs-FLP*^*1*^*UAS-cd8GFP/+* or *Y; tubP-Gal80 FRT40A/OK371-Gal4 FRT40A*. F, G, H, I: projections; adjacent panels: single optical sections. (F–F") Lineage 20–22 clone with a thin laterally (l) extending axon bundle (small arrow). Most postmitotic progeny (arrowheads), but not the neuroblast (arrows) express Oli. Asterisks indicate Oli-positive neurons adjacent to the clone; m, medial. (G–H") Lineage 15 clones, whose neurons extend a thick axon bundle laterally, defasciculate locally (asterisk) and exit the VNC (small arrow). Arrowheads show smaller later-born Oli-positive offspring, double arrowheads earlier-born larger Oli-negative neurons. (I–I") Lineage 24 clone, consisting of about 6 neurons, which extend axons laterally to exit the VNC. These neurons are Oli-negative (double arrowheads). Scale bars 20 μm.

Examining VNCs of wild-type 3rd instar larvae (Fig. 8A), we observed that many Oli-positive cells were organized in distinct lateral, intermediate and medial domains within thoracic neuromeres (Fig. 8B and C). Similar to embryonic stages, co-labeling with Mira and Repo revealed that Oli is primarily expressed in postmitotic neurons, but not in NBs and glia of postembryonic VNCs (Fig. 8D–E"'). At least five postembryonic thoracic NB lineages are known to generate motoneurons (lineages 15, 20–22, 24), which in adults innervate muscles within the different leg segments in a sterotypic pattern (Baek and Mann, 2009; Brierley et al., 2009; Brierley et al., 2012; Brown and Truman, 2009; Truman et al., 2004). A number of Oli-positive clones visualized with MARCM in conjunction with *elav-Gal4*^*c155*^ extended axons leaving the VNC, thus representing motoneuron-generating lineages (*n*=31/172). 22 clones were identified as lineages 20–22, which in addition to many interneurons contain one or two motoneurons with laterally projecting axons (Fig. 8F–F"). Most postmitotic progeny were Oli-positive. Moreover, 9 clones shared the characteristic features of lineage 15 (Fig. 8G–G""), which is solely composed of about 28–30 motoneurons, exiting the CNS in a thick bundle projecting to leg imaginal discs ((Truman et al., 2004); lineage A in (Baek and Mann, 2009)). *OK371-Gal4*, an enhancer trap insertion upstream of the vesicular glutamate transporter encoding gene *vGlut*, is specifically expressed in glutamergic neurons during embryonic and postembryonic development, as well as in adults (Baek and Mann, 2009; Brierley et al., 2009; Brierley et al., 2012; Brown and Truman, 2009; Mahr and Aberle, 2006). Analysis of MARCM clones labeled with this driver confirmed that Oli is present in lineage 15 motoneurons (*n*=9; Fig. 8H–H"). In these clones, the younger and slightly smaller progeny expressed Oli, while the larger, older neurons close to axon bundles were mostly Oli-negative (Fig. 8G–H"), suggesting a possible role in subtype specification or connectivity. In contrast, lineage 24 clones consisting of approximately 6 cells (Brown and Truman, 2009; Brierley et al., 2012) did not express Oli (*n*=5; Fig. 8I and I'). Oli expression is maintained in the adult VNC in *OK371-Gal4*-positive clusters, that comprise leg motoneurons (Baek and Mann, 2009) (Fig. S4B–B"). Hence, Oli is expressed in distinct postembryonic neuron lineages that include leg-innervating glutamatergic motoneurons.

### oli is required in lineages that include glutamatergic motoneurons

**Fig. S5:**
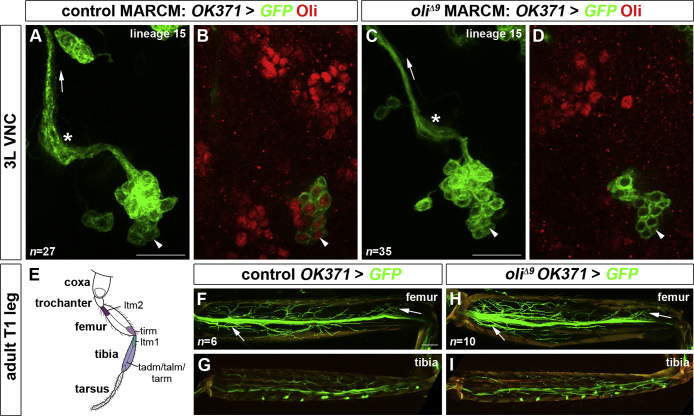
Assessment of oli function in controlling postembryonic lineage 15 development and axonal projections in the prothoracic leg. (A, B) *OK371-Gal4 MARCM: hs-FLP^1^ UAS-cd8GFP*/+ or *Y; tubP-Gal80 FRT40A/OK371-Gal4 FRT40A*. (C, D) *OK371-Gal4oli^Δ9^* MARCM: *hs-FLP^1^ UAS-cd8GFP*/+ or *Y; tubP-Gal80 FRT40A/OK371-Gal4oli^Δ9^FRT40A*. 3^rd^ instar larval control and oli deficient lineage 15 clones are of similar size suggesting that *oli* does not control clone proliferation. Neurons extend thick axon bundles laterally, defasciculate locally (asterisks) and exit the VNC (small arrow). Arrowheads show smaller later-born Oli-positive offspring (red) in controls, which also form normally in the absence of oli (arrow in D). Scale bar 20 μm. (E) Schematic of adult prothoracic leg segments consisting of coxa, trochanter, femur, tibia and five tarsi. Lineage 15 motoneurons innervate the long tendon (ltm2) and reductor muscle (tirm) groups in the femur, as well as the long tendon (ltm1), the tarsal depressor (tadm), tarsal levator (talm) and tarsal reductor muscles (tarm) in the tibia (adapted from (Brierley et al., 2012)). (F-I) Live images of adult prothoracic legs expressing membrane-bound GFP under the control of *OK371-Gal4*. (F, G) *OK371-Gal4 FRT40A*/*oli^Δ9^FRT40A; UAS-cd8GFP*. (H and I) *OK371-Gal4oli^Δ9^FRT40A*/*oli^Δ9^FRT40A; UAS-cd8GFP*. Motoneuron axons branch widely in the femur and tibia of controls (F and G) and *oli* mutant animals (H and I). Innervation patterns of tlm2 and tirm muscles in the femur are similar (arrows) (F, H). Scale bar 50 μm.

**Fig. 9:**
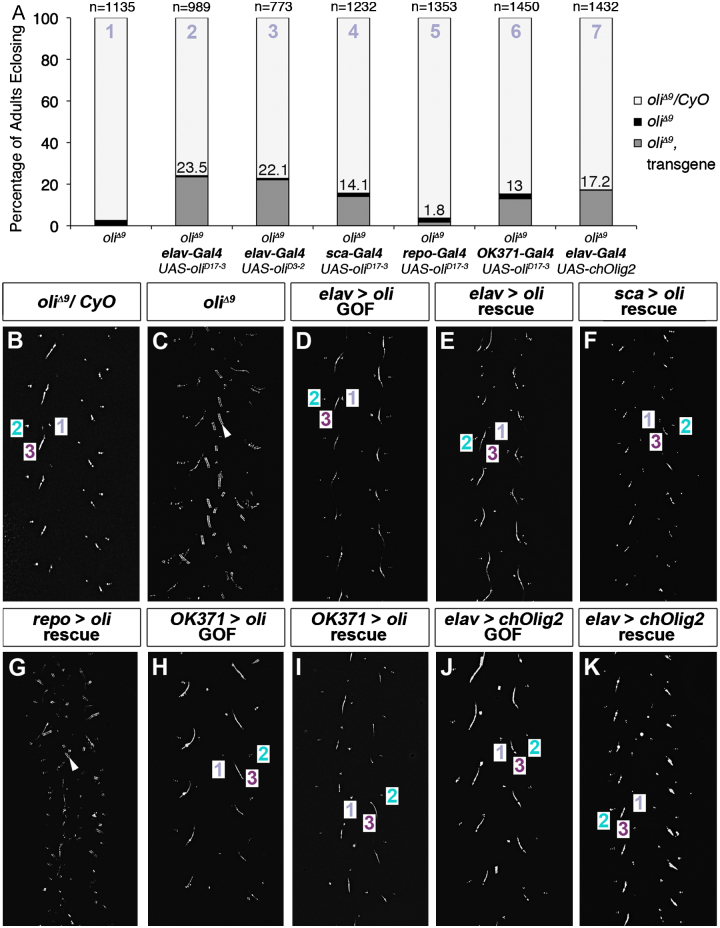
*oli* is required in glutamatergic neurons. (A) Quantification of adult eclosion rates. Percentages were determined for progeny: (i) heterozygous for *oli*^*Δ9*^, (ii) homozygous for *oli*^*Δ9*^ lacking the rescuing transgene, (iii) homozygous for *oli*^*Δ9*^ and containing the Gal4 driver and transgene. Gal4 drivers, transgenes and numbers of flies monitored in crosses 1–7 are as indicated. (B–K) Gain-of-function (GOF) and rescue leg print assays; 1–3, leg imprints. (B) *oli*^*Δ9*^ heterozygous controls show a normal alternating tripod gait. (C) *oli*^*Δ9*^ escapers (*n*=10) produce irregular traces (*n*=10). (D and E) Flies over-expressing *oli* with *elav-Gal4*^*c155*^ produce normal traces (*n*=14), and the walking pattern is fully rescued (*n*=16). (F) Expression with *sca-Gal4* rescues the gait; traces appear slightly irregular (*n*=9). (G) Expression with *repo-Gal4* fails to rescue (*n*=8). (H and I) Expression with *OK371-Gal4* does not affect walking (*n*=8), and rescues the gait, albeit not perfectly (*n*=17). (J and K) Expression of chick *Olig2* with *elav-Gal4*^*c155*^ does not cause walking defects (*n*=9), and rescues the gait; imprints of 3rd legs appear shorter (*n*=11).

To gain insights into a potential postembryonic function of *oli*, we examined MARCM clones lacking *oli* within the 3rd instar larval VNC. Consistent with the observation that Oli is not expressed in NBs, and thus unlikely to control proliferation, *oli* deficient lineage 15 motoneuron clones were indistinguishable from control clones (Fig. S5A–D). We therefore turned to rescue experiments monitoring adult eclosion and walking. In the postembryonic VNC, *elav-Gal4*^*c155*^ is active in all NBs, GMCs and postmitotic neurons (Fig. S4C–C"). Compared to 3.4% of escapers in controls, 23.5% of adult flies, which were mutant for *oli* and carried the *UAS-oli*^*D17−3*^ and *elav-Gal4*^*c155*^ transgenes, hatched in rescue crosses (Fig. 9A). The percentage is in the range of expected progeny (see Materials and methods), indicating that the eclosion rate was significantly restored. This was confirmed with a second independent transgene (*UAS-oli*^*D3−2*^; Fig. 9A). Oli over-expression did not cause discernible walking defects on its own and rescued deficits in an *oli*^*Δ9*^ background (Fig. 9B–E). Expression in a subset of postmitotic neurons using *sca-Gal4* (Fig. S4D–D") significantly restored eclosion rates (14.1%) and improved walking behavior (Fig. 9A and F). However consistent with the lack of expression in glia, ectopic expression using *repo-Gal4* neither rescued eclosion rates nor the walking defects of *oli*^*Δ9*^ adults (Fig. 9A and G). Furthermore, we used *OK371*-Gal4 to assess the requirement of *oli* in glutamatergic lineages. In accordance with our finding that Oli is not expressed exclusively in glutamatergic neurons (Fig. S4A–A"), we observed a partial, but nevertheless substantial rescue of the adult hatching rate (13%) and walking gait (Fig. 9A, H, I). Comparison of the overall muscle innervation pattern by *OK371-Gal4* positive motoneurons in prothoracic adult legs failed to uncover conspicuous defects in *oli* mutants compared to controls at this level of resolution (Fig. S5E–I). Finally, as *Drosophila* Oli and vertebrate Olig2 share 81% sequence identity within the bHLH domain (Fig. 1A), and Olig2 controls motoneuron formation (Briscoe and Novitch, 2008), we performed cross-species rescue experiments using a *UAS-chickOlig2* transgene. Intriguingly, expression of chick Olig2 with *elav-Gal4*^*c155*^ did not cause any locomotion defects, and considerably rescued adult hatching rates (17.2%), as well as the walking gait in the absence of fly *oli* (Fig. 9A, J, K).

These findings suggest that the lethality, as well as locomotion defects in adult escapers can at least partially be attributed to a functional requirement of *oli* in postembryonic neuronal lineages that include glutamatergic motoneurons, but not in glia. Moreover, *Drosophila* Oli may share some functional similarity with Olig2 in regulating the formation of neural circuits that control locomotion, possibly by interacting with or acting upstream of similar molecular determinants.

## Discussion

In this study, we provide functional evidence that *Drosophila oli* plays a pivotal role in the conserved transcriptional regulatory network governing motoneuron development, although the distinct steps requiring its activity differ in flies and vertebrates. Oli protein is mainly expressed in postmitotic neurons, as well as in some GMCs during embryonic development. This is consistent with in situ hybridization labeling detecting high levels of *oli* mRNA in postmitotic progeny, in addition to transient expression in MP2 and 7.1 NBs (Zhang et al., 2008). Oli is also expressed in postmitotic progeny of postembryonic lineages. By contrast, vertebrate Olig2 is required in progenitors to promote commitment to a general motoneuron identity (Mizuguchi et al., 2001). Also Olig1 and 3 largely function in progenitors (Lu et al., 2000; Muller et al., 2005; Takebayashi et al., 2000; Zechner et al., 2007; Zhou et al., 2000). Interestingly, Bhlhb4 and Bhlhb5 are expressed and required in postmitotic progeny of the retina, brain and spinal cord (Bramblett et al., 2004; Feng et al., 2006; Joshi et al., 2008; Liu et al., 2007). Thus, with respect to its primarily postmitotic expression, Drosophila Oli resembles more that of Bhlhb4 and Bhlhb5 than Olig1–3 in vertebrates.

The dynamic expression of *Drosophila* Oli is not consistent with that of a member of the temporal series of transcriptional regulators (Brody and Odenwald, 2000; Isshiki et al., 2001). With the latter, neurons largely maintain the determinant they expressed at the time of their birth. By contrast, Oli is widely expressed in newly born progeny, but subsequently levels decrease, and only some subtypes show high expression during late stages. Vertebrate *Olig2* acts as a transcriptional repressor in homomeric and heteromeric complexes, and expression is downregulated in differentiating motoneurons to enable the activation of postmitotic determinants such as Hb9 by Lim3, Isl1/2 and Neurog2 (Lee et al., 2005; Ma et al., 2008). Strikingly in flies, Oli expression decreases in RP and lateral ISNb motoneurons during embryogenesis and prolonged high expression of Oli elicits muscle innervation defects, supporting the notion that Oli downregulation is critical for its function in some neurons. Oli could thus act in a dual mode to regulate the differentiation of neuronal subtypes. The first one may rely on downregulation and be a feature shared with vertebrate Olig2, the second one may require persistent activity, and possibly be a feature more in common with Bhlhb4 and Bhlhb5 family members.

Our findings indicate that *Drosophila* Oli, unlike vertebrate Olig2, does not act as a general early somatic motoneuron determinant. It rather contributes to shaping ventral motoneuron subtype development as part of a postmitotic transcriptional regulatory network in concert with *Drosophila* Lim3, Isl, Hb9 and Dfr (Broihier et al., 2004; Broihier and Skeath, 2002; Odden et al., 2002; Thor et al., 1999; Thor and Thomas, 1997). This notion is supported by our findings that (i) Oli is co-expressed in specific combinations with these determinants in differentiated ISNb and TN motoneuron subtypes; (ii) similar to other ventral determinants, *oli* mutant embryos display distinct axonal pathfinding and muscle targeting defects; (iii) *oli* does not act upstream of *hb9*, *isl, lim3* or *Dfr;* and (iv) *oli* and *hb9* genetically interact, as loss of both enhances phenotypes in ISNb axons. Because of the proximity of *oli*, *isl* and *lim3* genetic loci*,* it has so far not been possible to further extend these interaction assays. Some defects observed in *oli* mutants, such as failure to innervate the clefts of muscles 12/13 or aberrant contacts between ISNb and TN motoneurons are qualitatively similar to those observed in *isl, lim3*, *hb9* and *dfr*, while the phenotype of *isl-τ-myc-*positive neurons abnormally exiting the VNC via the SN branch appears characteristic for *oli*. Moreover, the connectivity phenotypes observed in *oli* gain-of-function experiments were not reminiscent of trajectories of other motoneuron subtypes. This suggests that although Oli is a member of the combinatorial code, unlike for instance Dfr (Certel and Thor, 2004), it does not act as a simple switch between fates. It may rather act in concert or partially redundantly with these other determinants in regulating the stepwise process of axon guidance to ensure robustness of trajectory selection.

Individual transcription factors within an ensemble may regulate different biological properties to tightly coordinate the differentiation and synaptic connectivity of a given neuron subtype. As Oli does not act upstream of Isl, Lim3, Hb9 and Dfr, it may control the expression of other yet to be identified transcription factors, or—similar to *dfr*, *Nkx6* and *eve* in *Drosophila* and *Bhlhb5* in mice*—*axon guidance determinants (Broihier et al., 2004; Certel and Thor, 2004; Labrador et al., 2005; Ross et al., 2012) or—as reported for Neurog2— cytoskeletal regulators (Hand et al., 2005; Heng et al., 2008). Examining Fasciclin 3, N-Cadherin, PlexinA, and Frazzled (Iwai et al., 1997; Winberg et al., 1998a; Winberg et al., 1998b), we could not discern any obvious altered expression in the absence of *oli* (JO, IS, unpublished observations). Future studies using approaches such as microarrays will thus be required to identify *oli* downstream targets that control subtype-specific axonal connectivity.

While the role of *oli* in controlling neuronal development linked to locomotion appears conserved in *Drosophila* and vertebrates, conservation does not extend to glia. Oli is neither expressed in glia during embryonic or postembryonic development, nor is it essential for basic glial formation in the embryonic VNC or required in glia for locomotion. This also applied to other parts of the nervous system, such as the 3rd instar larval visual system endowed with large glial diversity (Chotard and Salecker, 2007) (Fig. S4E–G). hlh-17, the *C. elegans* Oli homolog, is expressed in cephalic sheath glia in the brain, and interestingly in some motoneurons in the larval CNS (Yoshimura et al., 2008). However, as analysis of *hlh-17* mutants could not pinpoint any requirement in glial generation and differentiation possibly due to redundancy with related factors, the precise role of the worm homolog remains elusive. Although ensheathing glia can be found in both invertebrates and vertebrates, myelinating glia have so far only been identified in vertebrates (Li and Richardson, 2008). This raises the possibility that the glial requirement of vertebrate Olig family members could be secondary, and Olig2 may have been recruited to collaborate with additional transcriptional regulators to promote the formation of myelinating glia. Indeed, Olig2 promotes motoneuron development together with Neurog2, and subsequently collaborates with Nkx2.2 to enable the generation of oligodendrocyte precursors and differentiating offspring from newly formed, uncommitted pMN progenitors (Wu et al., 2006). Interestingly in cell-based assays, Oli can physically interact with the Nkx2.2 homolog Ventral nervous system defective (Vnd) (Zhang et al., 2008). Together with our observation that Oli is not essential for glial development, this suggests that the potential of these determinants to interact is evolutionarily conserved, while the steps depending on them diverged in flies and vertebrates.

The locomotion defects in *oli* mutant larvae are likely the consequence of embryonic wiring defects, whereas the adult phenotypes may be due to an additional or even sole postembryonic requirement. Unlike the so far identified widely expressed determinants Chinmo, Broad Complex or Castor in the postembryonic VNC (Maurange et al., 2008), Oli expression is restricted to distinct lineages. That these include motoneurons is supported by our observations that Oli is detected in postembryonic lineages 20–22 and 15, and expression overlaps with that of *OK371-Gal4* (Baek and Mann, 2009; Brierley et al., 2009; Brierley et al., 2012; Truman et al., 2004). Moreover, locomotion defects can be partially rescued by over-expressing *oli* in glutamatergic neurons with this driver. Our initial characterization raises many new questions regarding the specific postembryonic role of Oli. Because of the expression in lineage 15, future experiments will need to specifically test, whether *oli* contributes to consolidating motoneuron subtype identity by regulating dendritic arbor-formation or leg muscle innervations with single cell resolution. The wider expression of Oli and the partial rescue with *OK371-Gal4* further suggest a requirement of *oli* in lineages that are part of locomotion-mediating neural circuits beyond motoneurons. Because of the expression pattern and the severe walking defects of adult *oli* escapers, our observations open the door for future functional studies to unravel the mechanisms that shape neural circuits underlying adult locomotion.

## Appendix A supplementary Material

**Table S1**. Number of hemisegments per genotype examined for muscle innervation defects shown in main figures.

## Footnotes

Appendix A Supplementary data associated with this article can be found in the online version at doi:10.1016/j.ydbio.2012.06.027.

## References

[bib1] Agius E., Soukkarieh C., Danesin C., Kan P., Takebayashi H., Soula C., Cochard P. (2004). Converse control of oligodendrocyte and astrocyte lineage development by Sonic hedgehog in the chick spinal cord. Dev. Biol..

[bib2] Arber S., Han B., Mendelsohn M., Smith M., Jessell T.M., Sockanathan S. (1999). Requirement for the homeobox gene Hb9 in the consolidation of motor neuron identity. Neuron.

[bib3] Baek M., Mann R.S. (2009). Lineage and birth date specify motor neuron targeting and dendritic architecture in adult Drosophila. J. Neurosci..

[bib4] Beckervordersandforth R.M., Rickert C., Altenhein B., Technau G.M. (2008). Subtypes of glial cells in the Drosophila embryonic ventral nerve cord as related to lineage and gene expression. Mech. Dev..

[bib5] Berger C., Renner S., Luer K., Technau G.M. (2007). The commonly used marker ELAV is transiently expressed in neuroblasts and glial cells in the Drosophila embryonic CNS. Dev. Dyn..

[bib6] Bertrand N., Castro D.S., Guillemot F. (2002). Proneural genes and the specification of neural cell types. Nat. Rev. Neurosci..

[bib7] Bramblett D.E., Copeland N.G., Jenkins N.A., Tsai M.J. (2002). BHLHB4 is a bHLH transcriptional regulator in pancreas and brain that marks the dimesencephalic boundary. Genomics.

[bib8] Bramblett D.E., Pennesi M.E., Wu S.M., Tsai M.J. (2004). The transcription factor Bhlhb4 is required for rod bipolar cell maturation. Neuron.

[bib9] Brand A.H., Perrimon N. (1993). Targeted gene expression as a means of altering cell fates and generating dominant phenotypes. Development.

[bib10] Brierley D.J., Blanc E., Reddy O.V., Vijayraghavan K., Williams D.W. (2009). Dendritic targeting in the leg neuropil of Drosophila: the role of midline signalling molecules in generating a myotopic map. PLoS Biol..

[bib11] Brierley D.J., Rathore K., VijayRaghavan K., Williams D.W. (2012). Developmental origins and architecture of Drosophila leg motoneurons. J. Comp. Neurol..

[bib12] Briscoe J., Novitch B.G. (2008). Regulatory pathways linking progenitor patterning, cell fates and neurogenesis in the ventral neural tube. Philos. Trans. R. Soc. Lond. B Biol. Sci..

[bib13] Brody T., Odenwald W.F. (2000). Programmed transformations in neuroblast gene expression during Drosophila CNS lineage development. Dev. Biol..

[bib14] Broihier H.T., Kuzin A., Zhu Y., Odenwald W., Skeath J.B. (2004). Drosophila homeodomain protein Nkx6 coordinates motoneuron subtype identity and axonogenesis. Development.

[bib15] Broihier H.T., Skeath J.B. (2002). Drosophila homeodomain protein dHb9 directs neuronal fate via crossrepressive and cell-nonautonomous mechanisms. Neuron.

[bib16] Brown, H.L., Truman, J.W., 2009. Fine-tuning of secondary arbor development: the effects of the ecdysone receptor on the adult neuronal lineages of the Drosophila thoracic CNS. Development. 10.1242/dev.039859 PMC2739142 19710167

[bib17] Certel S.J., Thor S. (2004). Specification of Drosophila motoneuron identity by the combinatorial action of POU and LIM-HD factors. Development.

[bib18] Chotard C., Salecker I. (2007). Glial cell development and function in the Drosophila visual system. Neuron Glia Biol..

[bib19] Chou T.B., Perrimon N. (1996). The autosomal FLP-DFS technique for generating germline mosaics in Drosophila melanogaster. Genetics.

[bib20] Crisp S., Evers J.F., Fiala A., Bate M. (2008). The development of motor coordination in Drosophila embryos. Development.

[bib21] di Sanguinetto Dalla Torre, Dasen S.A., Arber, S. J.S. (2008). Transcriptional mechanisms controlling motor neuron diversity and connectivity. Curr. Opin. Neurobiol..

[bib22] Dasen J.S. (2009). Transcriptional networks in the early development of sensory-motor circuits. Curr. Top Dev. Biol..

[bib23] Dessaud E., Yang L.L., Hill K., Cox B., Ulloa F., Ribeiro A., Mynett A., Novitch B.G., Briscoe J. (2007). Interpretation of the sonic hedgehog morphogen gradient by a temporal adaptation mechanism. Nature.

[bib24] Ding L., Takebayashi H., Watanabe K., Ohtsuki T., Tanaka K.F., Nabeshima Y., Chisaka O., Ikenaka K., Ono K. (2005). Short-term lineage analysis of dorsally derived Olig3 cells in the developing spinal cord. Dev. Dyn..

[bib25] Dixit R., Vijayraghavan K., Bate M. (2008). Hox genes and the regulation of movement in Drosophila. Dev. Neurobiol..

[bib26] Egger B., Chell J.M., Brand A.H. (2008). Insights into neural stem cell biology from flies. Philos. Trans. R. Soc. Lond. B Biol. Sci..

[bib27] Feng L., Xie X., Joshi P.S., Yang Z., Shibasaki K., Chow R.L., Gan L. (2006). Requirement for Bhlhb5 in the specification of amacrine and cone bipolar subtypes in mouse retina. Development.

[bib28] Freeman M.R., Doherty J. (2006). Glial cell biology in Drosophila and vertebrates. Trends Neurosci..

[bib29] Garces A., Bogdanik L., Thor S., Carroll P. (2006). Expression of Drosophila BarH1-H2 homeoproteins in developing dopaminergic cells and segmental nerve a (SNa) motoneurons. Eur. J. Neurosci..

[bib30] Garces A., Thor S. (2006). Specification of Drosophila aCC motoneuron identity by a genetic cascade involving even-skipped, grain and zfh1. Development.

[bib31] Godoy-Herrera R. (1986). The development and genetics of digging behavior in Drosophila larvae. Heredity.

[bib32] Gorczyca M.G., Phillis R.W., Budnik V. (1994). The role of tinman, a mesodermal cell fate gene, in axon pathfinding during the development of the transverse nerve in Drosophila. Development.

[bib33] Grosskortenhaus R., Pearson B.J., Marusich A., Doe C.Q. (2005). Regulation of temporal identity transitions in Drosophila neuroblasts. Dev. Cell.

[bib34] Halter D.A., Urban J., Rickert C., Ner S.S., Ito K., Travers A.A., Technau G.M. (1995). The homeobox gene repo is required for the differentiation and maintenance of glia function in the embryonic nervous system of Drosophila melanogaster. Development.

[bib35] Hand R., Bortone D., Mattar P., Nguyen L., Heng J.I., Guerrier S., Boutt E., Peters E., Barnes A.P., Parras C., Schuurmans C., Guillemot F., Polleux F. (2005). Phosphorylation of Neurogenin2 specifies the migration properties and the dendritic morphology of pyramidal neurons in the neocortex. Neuron.

[bib36] Heng J.I., Nguyen L., Castro D.S., Zimmer C., Wildner H., Armant O., Skowronska-Krawczyk D., Bedogni F., Matter J.M., Hevner R., Guillemot F. (2008). Neurogenin 2 controls cortical neuron migration through regulation of Rnd2. Nature.

[bib37] Ikeshima-Kataoka H., Skeath J.B., Nabeshima Y., Doe C.Q., Matsuzaki F. (1997). Miranda directs Prospero to a daughter cell during Drosophila asymmetric divisions. Nature.

[bib38] Isshiki T., Pearson B., Holbrook S., Doe C.Q. (2001). Drosophila neuroblasts sequentially express transcription factors which specify the temporal identity of their neuronal progeny. Cell.

[bib39] Iwai Y., Usui T., Hirano S., Steward R., Takeichi M., Uemura T. (1997). Axon patterning requires DN-cadherin, a novel neuronal adhesion receptor, in the Drosophila embryonic CNS. Neuron.

[bib40] Joshi P.S., Molyneaux B.J., Feng L., Xie X., Macklis J.D., Gan L. (2008). Bhlhb5 regulates the postmitotic acquisition of area identities in layers II-V of the developing neocortex. Neuron.

[bib41] Kambadur R., Koizumi K., Stivers C., Nagle J., Poole S.J., Odenwald W.F. (1998). Regulation of POU genes by castor and hunchback establishes layered compartments in the Drosophila CNS. Genes Dev..

[bib42] Klaes A., Menne T., Stollewerk A., Scholz H., Klambt C. (1994). The Ets transcription factors encoded by the Drosophila gene pointed direct glial cell differentiation in the embryonic CNS. Cell.

[bib43] Kosman D., Small S., Reinitz J. (1998). Rapid preparation of a panel of polyclonal antibodies to Drosophila segmentation proteins. Dev. Genes Evol..

[bib44] Labrador J.P., O'Keefe D., Yoshikawa S., McKinnon R.D., Thomas J.B., Bashaw G.J. (2005). The homeobox transcription factor even-skipped regulates netrin-receptor expression to control dorsal motor-axon projections in Drosophila. Curr. Biol..

[bib45] Landgraf M., Bossing T., Technau G.M., Bate M. (1997). The origin, location, and projections of the embryonic abdominal motorneurons of Drosophila. J. Neurosci..

[bib46] Landgraf M., Roy S., Prokop A., VijayRaghavan K., Bate M. (1999). even-skipped determines the dorsal growth of motor axons in Drosophila. Neuron.

[bib47] Landgraf M., Thor S. (2006). Development and structure of motoneurons. Int. Rev. Neurobiol..

[bib48] Landgraf M., Thor S. (2006). Development of Drosophila motoneurons: specification and morphology. Semin. Cell. Dev. Biol..

[bib49] Layden M.J., Odden J.P., Schmid A., Garces A., Thor S., Doe C.Q. (2006). Zfh1, a somatic motor neuron transcription factor, regulates axon exit from the CNS. Dev. Biol..

[bib50] Ledent V., Vervoort M. (2001). The basic helix-loop-helix protein family: comparative genomics and phylogenetic analysis. Genome. Res..

[bib51] Lee S.K., Jurata L.W., Funahashi J., Ruiz E.C., Pfaff S.L. (2004). Analysis of embryonic motoneuron gene regulation: derepression of general activators function in concert with enhancer factors. Development.

[bib52] Lee S.K., Lee B., Ruiz E.C., Pfaff S.L. (2005). Olig2 and Ngn2 function in opposition to modulate gene expression in motor neuron progenitor cells. Genes Dev..

[bib53] Lee S.K., Pfaff S.L. (2003). Synchronization of neurogenesis and motor neuron specification by direct coupling of bHLH and homeodomain transcription factors. Neuron.

[bib54] Lee T., Luo L. (1999). Mosaic analysis with a repressible cell marker for studies of gene function in neuronal morphogenesis. Neuron.

[bib55] Li H., Richardson W.D. (2008). The evolution of Olig genes and their roles in myelination. Neuron Glia Biol..

[bib56] Ligon K.L., Fancy S.P., Franklin R.J., Rowitch D.H. (2006). Olig gene function in CNS development and disease. Glia.

[bib57] Liu B., Liu Z., Chen T., Li H., Qiang B., Yuan J., Peng X., Qiu M. (2007). Selective expression of Bhlhb5 in subsets of early-born interneurons and late-born association neurons in the spinal cord. Dev. Dyn..

[bib58] Lu Q.R., Sun T., Zhu Z., Ma N., Garcia M., Stiles C.D., Rowitch D.H. (2002). Common developmental requirement for Olig function indicates a motor neuron/oligodendrocyte connection. Cell.

[bib59] Lu Q.R., Yuk D., Alberta J.A., Zhu Z., Pawlitzky I., Chan J., McMahon A.P., Stiles C.D., Rowitch D.H. (2000). Sonic hedgehog--regulated oligodendrocyte lineage genes encoding bHLH proteins in the mammalian central nervous system. Neuron.

[bib60] Ma Y.C., Song M.R., Park J.P., Henry Ho, H.Y., Hu L., Kurtev M.V., Zieg J., Ma Q., Pfaff S.L., Greenberg M.E. (2008). Regulation of motor neuron specification by phosphorylation of neurogenin 2. Neuron.

[bib61] Mahr A., Aberle H. (2006). The expression pattern of the Drosophila vesicular glutamate transporter: a marker protein for motoneurons and glutamatergic centers in the brain. Gene Expr. Patterns.

[bib62] Maqbool T., Soler C., Jagla T., Daczewska M., Lodha N., Palliyil S., VijayRaghavan K., Jagla K. (2006). Shaping leg muscles in Drosophila: role of ladybird, a conserved regulator of appendicular myogenesis. PLoS ONE.

[bib63] Maurange C., Cheng L., Gould A.P. (2008). Temporal transcription factors and their targets schedule the end of neural proliferation in Drosophila. Cell.

[bib64] Miguel-Aliaga I., Allan D.W., Thor S. (2004). Independent roles of the dachshund and eyes absent genes in BMP signaling, axon pathfinding and neuronal specification. Development.

[bib65] Mizuguchi R., Sugimori M., Takebayashi H., Kosako H., Nagao M., Yoshida S., Nabeshima Y., Shimamura K., Nakafuku M. (2001). Combinatorial roles of olig2 and neurogenin2 in the coordinated induction of pan-neuronal and subtype-specific properties of motoneurons. Neuron.

[bib66] Moore A.W., Barbel S., Jan L.Y., Jan Y.N. (2000). A genomewide survey of basic helix-loop-helix factors in Drosophila. Proc. Natl. Acad. Sci. USA.

[bib67] Muller T., Anlag K., Wildner H., Britsch S., Treier M., Birchmeier C. (2005). The bHLH factor Olig3 coordinates the specification of dorsal neurons in the spinal cord. Genes Dev..

[bib68] Novitch B.G., Chen A.I., Jessell T.M. (2001). Coordinate regulation of motor neuron subtype identity and pan-neuronal properties by the bHLH repressor Olig2. Neuron.

[bib69] Odden J.P., Holbrook S., Doe C.Q. (2002). Drosophila HB9 is expressed in a subset of motoneurons and interneurons, where it regulates gene expression and axon pathfinding. J. Neurosci..

[bib70] Ohshiro T., Yagami T., Zhang C., Matsuzaki F. (2000). Role of cortical tumour-suppressor proteins in asymmetric division of Drosophila neuroblast. Nature.

[bib71] Peyrefitte S., Kahn D., Haenlin M. (2001). New members of the Drosophila Myc transcription factor subfamily revealed by a genome-wide examination for basic helix-loop-helix genes. Mech. Dev..

[bib72] Robinow S., White K. (1991). Characterization and spatial distribution of the ELAV protein during Drosophila melanogaster development. J. Neurobiol..

[bib73] Ross S.E., Mardinly A.R., McCord A.E., Zurawski J., Cohen S., Jung C., Hu L., Mok S.I., Shah A., Savner E.M., Tolias C., Corfas R., Chen S., Inquimbert P., Xu Y., McInnes R.R., Rice F.L., Corfas G., Ma Q., Woolf C.J., Greenberg M.E. (2010). Loss of inhibitory interneurons in the dorsal spinal cord and elevated itch in Bhlhb5 mutant mice. Neuron.

[bib74] Ross S.E., McCord A.E., Jung C., Atan D., Mok S.I., Hemberg M., Kim T.K., Salogiannis J., Hu L., Cohen S., Lin Y., Harrar D., McInnes R.R., Greenberg M.E. (2012). Bhlhb5 and prdm8 form a repressor complex involved in neuronal circuit assembly. Neuron.

[bib75] Skaggs K., Martin D.M., Novitch B.G. (2011). Regulation of spinal interneuron development by the Olig-related protein Bhlhb5 and Notch signaling. Development.

[bib76] Skeath J.B., Thor S. (2003). Genetic control of Drosophila nerve cord development. Curr. Opin. Neurobiol..

[bib77] Suster M.L., Bate M. (2002). Embryonic assembly of a central pattern generator without sensory input. Nature.

[bib78] Takebayashi H., Ohtsuki T., Uchida T., Kawamoto S., Okubo K., Ikenaka K., Takeichi M., Chisaka O., Nabeshima Y. (2002). Non-overlapping expression of Olig3 and Olig2 in the embryonic neural tube. Mech. Dev..

[bib79] Takebayashi H., Yoshida S., Sugimori M., Kosako H., Kominami R., Nakafuku M., Nabeshima Y. (2000). Dynamic expression of basic helix-loop-helix Olig family members: implication of Olig2 in neuron and oligodendrocyte differentiation and identification of a new member, Olig3. Mech. Dev..

[bib80] Thor S., Andersson S.G., Tomlinson A., Thomas J.B. (1999). A LIM-homeodomain combinatorial code for motor-neuron pathway selection. Nature.

[bib81] Thor S., Thomas J.B. (1997). The Drosophila islet gene governs axon pathfinding and neurotransmitter identity. Neuron.

[bib82] Thor S., Thomas J.B. (2002). Motor neuron specification in worms, flies and mice: conserved and ‘lost’ mechanisms. Curr. Opin. Genet. Dev..

[bib83] Truman J.W., Schuppe H., Shepherd D., Williams D.W. (2004). Developmental architecture of adult-specific lineages in the ventral CNS of Drosophila. Development.

[bib84] Tsuchida T., Ensini M., Morton S.B., Baldassare M., Edlund T., Jessell T.M., Pfaff S.L. (1994). Topographic organization of embryonic motor neurons defined by expression of LIM homeobox genes. Cell.

[bib85] Van Vactor D.V., Sink H., Fambrough D., Tsoo R., Goodman C.S. (1993). Genes that control neuromuscular specificity in Drosophila. Cell.

[bib86] Winberg M.L., Mitchell K.J., Goodman C.S. (1998). Genetic analysis of the mechanisms controlling target selection: complementary and combinatorial functions of netrins, semaphorins, and IgCAMs. Cell.

[bib87] Winberg M.L., Noordermeer J.N., Tamagnone L., Comoglio P.M., Spriggs M.K., Tessier-Lavigne M., Goodman C.S. (1998). Plexin A is a neuronal semaphorin receptor that controls axon guidance. Cell.

[bib88] Wu S., Wu Y., Capecchi M.R. (2006). Motoneurons and oligodendrocytes are sequentially generated from neural stem cells but do not appear to share common lineage-restricted progenitors in vivo. Development.

[bib89] Xiong W.C., Okano H., Patel N.H., Blendy J.A., Montell C. (1994). repo encodes a glial-specific homeo domain protein required in the Drosophila nervous system. Genes Dev..

[bib90] Xu Z.P., Dutra A., Stellrecht C.M., Wu C., Piatigorsky J., Saunders G.F. (2002). Functional and structural characterization of the human gene BHLHB5, encoding a basic helix-loop-helix transcription factor. Genomics.

[bib91] Yoshimura S., Murray J.I., Lu Y., Waterston R.H., Shaham S. (2008). mls-2 and vab-3 Control glia development, hlh-17/Olig expression and glia-dependent neurite extension in *C. elegans*. Development.

[bib92] Zechner D., Muller T., Wende H., Walther I., Taketo M.M., Crenshaw E.B., Treier M., Birchmeier W., Birchmeier C. (2007). Bmp and Wnt/beta-catenin signals control expression of the transcription factor Olig3 and the specification of spinal cord neurons. Dev. Biol..

[bib93] Zhang H., Syu L.J., Modica V., Yu Z., Von Ohlen T., Mellerick D.M. (2008). The Drosophila homeodomain transcription factor, Vnd, associates with a variety of co-factors, is extensively phosphorylated and forms multiple complexes in embryos. FEBS J..

[bib94] Zhou Q., Anderson D.J. (2002). The bHLH transcription factors OLIG2 and OLIG1 couple neuronal and glial subtype specification. Cell.

[bib95] Zhou Q., Choi G., Anderson D.J. (2001). The bHLH transcription factor Olig2 promotes oligodendrocyte differentiation in collaboration with Nkx2.2. Neuron.

[bib96] Zhou Q., Wang S., Anderson D.J. (2000). Identification of a novel family of oligodendrocyte lineage-specific basic helix-loop-helix transcription factors. Neuron.

