# Supplementary material for: Regulation of locomotion and motoneuron trajectory selection and targeting by the Drosophila homolog of Olig family transcription factors
Source: Dev Biol. 2012 Sep 15;369(2-2):261–76. doi: 10.1016/j.ydbio.2012.06.027 (PMC3464432; doi:10.1016/j.ydbio.2012.06.027)
Supplement: Supplementary file 5 — Table S1. Number of hemisegments per genotype examined for muscle innervation defects shown in main figures. [file mmc10.doc]

**Table S1.** Number of hemisegments per genotype examined for muscle innervation defects.

| Genotype | Hemisegments  n= | Hemisegments with projection defects  n= | | | |
| --- | --- | --- | --- | --- | --- |
|  | Total | Total^1^ | Phenotype 1:  ISNb | Phenotype 2:  ISNb/TN | Phenotype 3:  TN/LBD |
| wild type | 124 | 13 | 6 | 3 | 4 |
| *oli^Δ9^* | 176 | 91 | 34 | 49 | 25 |
| *elav-Gal4^c155^/+* or *Y; oli^Δ9^; UAS-oli/+* | 105 | 51 | 28 | 27 | 18 |
| *elav-Gal4^c155^/+* or *Y; UAS-oli/+* | 114 | 56 | 52 | 7 | 6 |
| *sca-Gal4, oli^Δ9^*/ *oli^Δ9^; UAS-oli/+* | 110 | 37 | 34 | 13 | 2 |
| *sca-Gal4/+; UAS-oli/+* | 86 | 24 | 22 | 2 | 3 |
| *hb9^KK30^/hb9^KK30^* | 94 | 66 ^2^ | 32 | 42 | 4 |
| *hb9^KK30^/hb9^Gal4^* | 72 | 48 ^2^ | 25 | 20 | 2 |
| *oli^Δ9^; hb9^KK30^* | 28 | 24 ^3^ | 19 | 11 | 2 |
| *oli^Δ9^; hb9^KK30^/hb9^Gal4^* | 26 | 25 ^3^ | 18 | 6 | 4 |

^1^ Total numbers of hemisegments displaying projection defects are lower than the sum of the three phenotype classes, as some hemisegments display more than one defect.

^2, 3^ Percentages of defects were calculated individually for each genotype. Bar graphs in Figure 6L show average percentages for *hb9* and *oli^Δ9^; hb9* single and double mutant values.
